# Supplementary material for: Effect of enforcement of the national referral guidelines on patterns of orthopedic admissions to Kenyatta National Hospital, Kenya: Pre-post intervention study
Source: PLoS One. 2024 Aug 13;19(8):e0290195. doi: 10.1371/journal.pone.0290195 (PMC11321550; doi:10.1371/journal.pone.0290195)
Supplement: Attachment — Submitted filename: PONE-D-23-24470.pdf [file pone.0290195.s003.pdf]

PLOS ONE

Effect of enforcement of referral guidelines on patterns of orthopedic and trauma admissions to Kenyatta National Hospital, Kenya: pre-posttest study design

--Manuscript Draft--

**Manuscript Number:**	PONE-D-23-24470
**Article Type:**	Research Article
**Full Title:**	Effect of enforcement of referral guidelines on patterns of orthopedic and trauma admissions to Kenyatta National Hospital, Kenya: pre-posttest study design
**Short Title:**	effect of referral guidelines on orthopedic and trauma admissions to Kenyatta National Hospital
**Corresponding Author:**	Maxwell Philip Omondi, PhD<br>University of Nairobi College of Health Sciences: University of Nairobi Faculty of Health Sciences<br>Nairobi, KENYA
**Keywords:**	orthopedic and trauma admissions, Kenyatta National Hospital, referral guidelines
**Abstract:**	Inappropriate utilization of higher-level health facilities and ineffective management of referral processes in resource-limited settings is increasingly becoming a concern in health care management in developing countries. This is characterized by self-referral and frequent bypassing of the nearest health facilities coupled with low formal referral mechanisms. This scenario lends itself to a situation where uncomplicated medical conditions are unnecessarily managed in a high-cost health facility. On July 1, 2021, Kenyatta National Hospital (KNH) did enforce the Kenya Health Sector Referral Implementation Guidelines, 2014 which required patients get approval from KNH referral office and a formal referral letter to be admitted at KNH to reduce the number of walk-ins and allow KNH to function as a referral facility as envisioned by Kenya 2010 Constitution and KNH legal statue of 1987. The study objective was to determine the effect of enforcement of referral guidelines on patterns of orthopaedic and trauma admissions to KNH. This was a pre-post-test study design. Data abstraction was done for 459 and 446 charts before and after enforcement of referral guidelines. Enforcement of the referral guidelines reduced the proportion of walk-in admissions from 54.9% to 45.1%, while proportion of facility referrals increased from 46.6% to 53.4% (p=0.013). The Non-trauma orthopaedic admissions doubled from 12.0% to 22.4% (p<0.001). There was also a notable increase in admissions through the Outpatient Clinic (OC) and Corporate Outpatient Clinic (COC). The proportion of emergency admissions declined while that of elective admissions increased. The increase in elective cases were mainly driven by the increase in female admissions with active insurance cover, tertiary education, non-trauma related conditions and of older age groups. However, use of official formal written referral letters did not change despite the enforcement of referral guidelines. In conclusion, the enforcement of the referral guidelines reduced the proportion of walk-ins' admissions to KNH. The admissions were more non-trauma conditions, had tertiary education level, and more active insurance cover as a result of enforcement of the referral guidelines.
**Order of Authors:**	Maxwell Philip Omondi, PhD
	Joseph Chege Mwangi, MD Orthopedic Surgery
	Fred Chuma Sitati, MD Orthopedic Surgery
	Herbert Philip Onga'ngo
**Additional Information:**	
**Question**	**Response**
**Financial Disclosure**<br><br>Enter a financial disclosure statement that describes the sources of funding for the	This study was partially funded by Kenyatta National Hospital (https://knh.or.ke/). MPO received the funding award. The research grant award was RFA 2020/21. The funders had no role in study design, data collection and analysis, decision to publish, or preparation of the manuscript.

Powered by Editorial Manager® and ProduXion Manager® from Aries Systems Corporation

work included in this submission. Review the submission guidelines for detailed requirements. View published research articles from *PLOS ONE* for specific examples.<br><br>This statement is required for submission and **will appear in the published article** if the submission is accepted. Please make sure it is accurate.<br><br>**Unfunded studies**<br>Enter: *The author(s) received no specific funding for this work.*<br><br>**Funded studies**<br>Enter a statement with the following details:<br>• Initials of the authors who received each award<br>• Grant numbers awarded to each author<br>• The full name of each funder<br>• URL of each funder website<br>• Did the sponsors or funders play any role in the study design, data collection and analysis, decision to publish, or preparation of the manuscript?<br>• **NO** - Include this sentence at the end of your statement: *The funders had no role in study design, data collection and analysis, decision to publish, or preparation of the manuscript.*<br>• **YES** - Specify the role(s) played.<br><br>* typeset	
**Competing Interests**<br><br>Use the instructions below to enter a competing interest statement for this submission. On behalf of all authors, disclose any competing interests that could be perceived to bias this work—acknowledging all financial support and any other relevant financial or non-financial competing interests.<br><br>This statement is required for submission and **will appear in the published article** if the submission is accepted. Please make sure it is accurate and that any funding sources listed in your Funding Information	The authors have declared that no competing interests exist.

later in the submission form are also declared in your Financial Disclosure statement.<br><br>View published research articles from *PLOS ONE* for specific examples.<br><br>**NO authors have competing interests**<br><br>Enter: *The authors have declared that no competing interests exist.*<br><br>**Authors with competing interests**<br><br>Enter competing interest details beginning with this statement:<br><br>*I have read the journal's policy and the authors of this manuscript have the following competing interests: [insert competing interests here]*<br><br>* typeset	
**Ethics Statement**<br><br>Enter an ethics statement for this submission. This statement is required if the study involved:<br><br>• Human participants<br>• Human specimens or tissue<br>• Vertebrate animals or cephalopods<br>• Vertebrate embryos or tissues<br>• Field research<br><br>Write "N/A" if the submission does not require an ethics statement.<br><br>General guidance is provided below. Consult the submission guidelines for detailed instructions. **Make sure that all information entered here is included in the Methods section of the manuscript.**	N/A

**Format for specific study types**<br><br>**Human Subject Research (involving human participants and/or tissue)**<br>• Give the name of the institutional review board or ethics committee that approved the study<br>• Include the approval number and/or a statement indicating approval of this research<br>• Indicate the form of consent obtained (written/oral) or the reason that consent was not obtained (e.g. the data were analyzed anonymously)<br><br>**Animal Research (involving vertebrate animals, embryos or tissues)**<br>• Provide the name of the Institutional Animal Care and Use Committee (IACUC) or other relevant ethics board that reviewed the study protocol, and indicate whether they approved this research or granted a formal waiver of ethical approval<br>• Include an approval number if one was obtained<br>• If the study involved *non-human primates*, add *additional details* about animal welfare and steps taken to ameliorate suffering<br>• If anesthesia, euthanasia, or any kind of animal sacrifice is part of the study, include briefly which substances and/or methods were applied<br><br>**Field Research**<br><br>Include the following details if this study involves the collection of plant, animal, or other materials from a natural setting:<br>• Field permit number<br>• Name of the institution or relevant body that granted permission	
**Data Availability**<br><br>Authors are required to make all data underlying the findings described fully available, without restriction, and from the time of publication. PLOS allows rare exceptions to address legal and ethical concerns. See the PLOS Data Policy and FAQ for detailed information.	Yes - all data are fully available without restriction

A Data Availability Statement describing where the data can be found is required at submission. Your answers to this question constitute the Data Availability Statement and **will be published in the article**, if accepted.<br><br>**Important:** Stating 'data available on request from the author' is not sufficient. If your data are only available upon request, select 'No' for the first question and explain your exceptional situation in the text box.<br><br>Do the authors confirm that all data underlying the findings described in their manuscript are fully available without restriction?	
**Describe where the data may be found in full sentences. If you are copying our sample text, replace any instances of XXX with the appropriate details.**<br><br>• If the data are **held or will be held in a public repository**, include URLs, accession numbers or DOIs. If this information will only be available after acceptance, indicate this by ticking the box below. For example: *All XXX files are available from the XXX database (accession number(s) XXX, XXX.)*.<br>• If the data are all contained **within the manuscript and/or Supporting Information files**, enter the following: *All relevant data are within the manuscript and its Supporting Information files.*<br>• If neither of these applies but you are able to provide **details of access elsewhere**, with or without limitations, please do so. For example:<br><br>*Data cannot be shared publicly because of [XXX]. Data are available from the XXX Institutional Data Access / Ethics Committee (contact via XXX) for researchers who meet the criteria for access to confidential data.*<br><br>*The data underlying the results presented in the study are available from (include the name of the third party*	All relevant data are within the manuscript and its Supporting Information files.

and contact information or URL).<br>• This text is appropriate if the data are owned by a third party and authors do not have permission to share the data.<br><br>* typeset	
Additional data availability information:	

Powered by Editorial Manager® and ProduXion Manager® from Aries Systems Corporation

Manuscript
Click here to access/download;Manuscript;manuscript _ Effect of guidelines.docx

Effect of enforcement of referral guidelines on patterns of orthopedic and trauma admissions to Kenyatta National Hospital, Kenya: pre-posttest study design

Authors: Maxwell Philip Omondi[1¶], Joseph Chege Mwangi[1¶], Fred Chuma Sitati[1¶], Herbert Onga'ngo'[2¶]

[1] Orthopedics Unit, Department of Surgery, University of Nairobi, Nairobi, Kenya
[2] Department of Orthopedics and Trauma, Kenyatta National Hospital, Nairobi, Kenya

*Corresponding author: Maxwell Philip Omondi
Email: maxwellomondi@gmail.com

¶All authors contributed equally to this work.

Abstract

Inappropriate utilization of higher-level health facilities and ineffective management of referral processes in resource-limited settings is increasingly becoming a concern in health care management in developing countries. This is characterized by self-referral and frequent bypassing of the nearest health facilities coupled with low formal referral mechanisms. This scenario lends itself to a situation where uncomplicated medical conditions are unnecessarily managed in a high-cost health facility. On July 1, 2021, Kenyatta National Hospital (KNH) did enforce the Kenya Health Sector Referral Implementation Guidelines, 2014 which required patients get approval from KNH referral office and a formal referral letter to be admitted at KNH to reduce the number of walk-ins

and allow KNH to function as a referral facility as envisioned by Kenya 2010 Constitution and KNH legal statue of 1987. The study objective was to determine the effect of enforcement of referral guidelines on patterns of orthopaedic and trauma admissions to KNH. This was a pre-post-test study design. Data abstraction was done for 459 and 446 charts before and after enforcement of referral guidelines. Enforcement of the referral guidelines reduced the proportion of walk-in admissions from 54.9% to 45.1%, while proportion of facility referrals increased from 46.6% to 53.4% ($p=0.013$). The Non-trauma orthopaedic admissions doubled from 12.0% to 22.4% ($p<0.001$). There was also a notable increase in admissions through the Outpatient Clinic (OC) and Corporate Outpatient Clinic (COC). The proportion of emergency admissions declined while that of elective admissions increased. The increase in elective cases were mainly driven by the increase in female admissions with active insurance cover, tertiary education, non-trauma related conditions and of older age groups. However, use of official formal written referral letters did not change despite the enforcement of referral guidelines. In conclusion, the enforcement of the referral guidelines reduced the proportion of walk-ins' admissions to KNH. The admissions were more non-trauma conditions, had tertiary education level, and more active insurance cover as a result of enforcement of the referral guidelines.

Keys: referral guidelines, Orthopaedic and trauma, pattern of admission

Background

Approximately 90% of the estimated traumatic injuries occur in low and middle-income countries according to World Health Organization (1) and this represents an important global public health problem now and in the coming years (2, 3).

Tertiary hospitals in resource-limited countries treat referred patients but in most cases are the first level of care for the vast majority of patients (4). One of the challenges in health care delivery in resource-limited settings is the inappropriate utilization of tertiary health facilities that results in patients' congestion in referral hospitals with simple conditions that can be effectively managed at lower health facilities. The majority of these patients are self-referred, bypassing lower-level health facilities in the process (5-8).

This jeopardizes the appropriate delivery of primary, secondary, and tertiary health care. Tertiary health care is compromised by the large demand for primary health care and in the process cripples the primary health care system and this effectively ensures that primary health care facilities remain underused and inefficient (9).

Kenya is administratively organized into forty-seven (47) counties with Nairobi Capital city being in Nairobi County. Health care service delivery has been devolved to the county level with the national level being responsible for policy formulation and strategic planning. KNH falls under national government administratively(10). The Kenya health service delivery system is organized across six levels of care, beginning at the community level and continuing through primary care services, which include dispensaries (level 2)

and health centers (level 3), and county referral health services (level 4 & 5) all the way to the national referral health services (level 6) (See Fig 1)(11). KNH is a level 6 facility which means it is a national tertiary level hospital in the Kenyan health system that offers very specialized skills, expertise and services(11).

Figure 1: referral linkages between different levels of care (Reproduced from Kenya Health Sector Implementation Referral Guidelines, 2014)

Kenyatta National Hospital (KNH) was established in 1901 as a National Referral and Teaching Hospital to provide training and medical research in Kenya. KNH became a State Corporation in 1987 and sits at the apex of the health referral system in Kenya (12). According to the KNH Board order of 1987 contained in the Legal Notice No. 109, the functions of KNH were spelled out as a) to receive patients on referral for specialized health care; b) to provide facilities for medical education for the University of Nairobi and other health allied courses; c) to contribute to national health planning (12). This understanding has been reinforced by the Kenya Health Sector Referral Implementation Guidelines, 2014, and the Constitution of Kenya 2010 which tasks KNH with the responsibility for health policy formulation (10, 11).

Orthopaedic wards in KNH have consistently recorded the highest bed occupancy percent for the last couple of years. In 2018, 2019, and 2020, it recorded bed occupancy percent of 142.2%, 138.2%, and 116.5%, respectively, with the overall KNH bed

occupancy percent of 106.2%, 113.4 percent and 91.5% (13). The consequence of this is the low nurse-patient ratio of 1:10 that compromises not only the quality of nursing care given to patients but also compromises the ability of KNH to effectively perform its statutory obligations.

At the strategic level, the government's overall strategic plan, the Second Medium-Term Plan (MTP) of Vision 2030, identifies enforcement of referral guidelines as a key strategy to increase the use of services at lower levels of the health care system and reduce self-referral to higher levels of care.

Consequently, on 1st July 2021, KNH management made a decision to reinforce the Kenya Health Sector Referral Guidelines 2014 that places KNH at the tip of the health sector referral system. This meant that patients will be seen based on referral letters from other health facilities to reduce the number of walk-in patients who would have otherwise been appropriately seen at the peripheral health facilities. This will contribute to the decongestion of KNH Orthopaedic and trauma admissions. The purpose of this study was to determine the effect of the enforcement of referral guidelines on the patterns of orthopaedic and trauma admissions to Kenyatta National Hospital.

Methods and Materials

Study design: This was a pre-post-test study design. Enforcement of referral guidelines required that the referring health facility consults with KNH referral coordination Office for concurrence before that patients are referred to KNH. This is to ensure only a) patients

who require specialized medical and orthopaedic and trauma care or b) no neighbouring government health facility has the capacity to manage the cases, get admitted to KNH.

Study area: KNH is the largest teaching and referral hospital in East and Central Africa. KNH Orthopaedic Wards were the study area. KNH is based in Upperhill, Nairobi, the capital city of Kenya. It is located along Hospital Road, about 5km from the city centre. It has a bed capacity of approximately 2000 beds.

Study population: Orthopaedic in-patient case loads

Study duration: The study duration was from February 1st to December 31st 2021. It covered 5 months before and 5 months after enforcement of the referral guidelines, that is, from February 1, to June 30, 2021, before enforcement of the referral guidelines and from August 1, to December 31, 2021 after enforcement of the referral guidelines. The referral guidelines were enforced from July 1, 2021. Inclusion criteria was all orthopaedic and trauma admissions to KNH during the study period. 1st February to 31st December 2021. Data was accessed from the patient charts from 1st January to 31st March 2022 during the data collection period.

Sample size calculation and sampling procedure: Using an adjusted formula for calculating sample sizes that compare two binomial distributions (14), a total of 459 files and 446 files were abstracted before and after the enforcement of the referral guidelines. Stratified sampling technique was used with orthopaedic and trauma admissions being stratified by month of admission and Population Proportionate to Size (PPS) was used to determine the sample size per strata. Systematic random sampling was then used to identify the sampled files using the KNH Health Records Register as the sampling frame from each of these three service entry points {Accident and Emergency (A&E),

Orthopedic Clinic (OC), and Corporate Outpatient Clinic (COC)}. This ensured the selected sample would be representative of the admissions by month from each of these three orthopedic and trauma admissions entry points. Informed consent, auditory, and visual confidentiality were provided.

Variables: variables abstracted for this study were a) Socio-demographic characteristics – age, sex, marital status, religion, education level b) Nature of admission; c) Occupation; d) Type of admission; e) Mode of payment;

Data collection procedures: Data collection was done through a data abstraction form.

Quality assurance & quality control procedures: Three Research assistants underwent two (2) day training on the study protocol. A pilot study was conducted during the design of the study protocol to test the data collection tools for relevance, appropriateness to answer the research questions, and adjustments of the data collection tools made as necessary. The Principal Investigator conducted daily reviews of all abstracted forms, verified for accuracy, completeness, and compliance with the research protocol.

Ethical considerations: UoN/KNH Ethics and Research Committee granted ethical approval (ERC No: P852/10/2021). Administrative approval was also sought from KNH Medical Research Department and KNH Orthopaedics Department. This study was carried out in accordance with UoN/KNH Ethics and Research Committee guidelines and regulations. For the underage, formal verbal consent was obtained from the parent/guardian.

Data management and analysis plan: Data abstraction tool was used to collect quantitative data. The data were entered into a password-protected Redcap database. Descriptive analysis was done using measures of central tendency (mean), measures of dispersions such as range, standard deviation were used, frequency distribution were also used. Inferential analysis was done using Pearsons's chi-square test, logistic regression, odds ratio to determine effect of the enforcement of the referral guidelines and also determine possible associations. SPSS version 21 was used.

Study limitations: a) Incomplete and missing records – this was addressed by increasing the sample size by 10%; c) Effect of COVID 19 pandemic on referrals of cases from peripheral health facilities and walk-in patients – this was addressed by ensuring the data collection period covered the covid period.

Results

Socio-demographic characteristics of the sample population

Key socio-demographic characteristics of orthopaedic and trauma admissions at KNH such as age, sex, education, occupation, and marital status were reviewed and compared between the two groups. Education and sex were noted to be statistically significant between the two groups at p=0.001 and p=0.005, respectively. However, age, religion, occupation, marital status, smoking, and alcohol intake habits were comparable amongst the two groups (P > 0.05) (Table 1).

Table 1: Socio-demographic characteristics between orthopaedic and trauma admissions at KNH before and after enforcement of the referral guidelines, 2021

Variable	Category	Before (n=459)	After (n=446)	Chi-square, p-value
Age	0 – 14 years	59 (59.6%)	40 (40.4%)	7.121 (p=0.068)
	15 – 24 years	83 (50.0%)	83 (50.0%)	
	25 – 64 years	303 (50.5%)	297 (49.5%)	

	Above 65 years	14 (35.0%)	26 (65.0%)	
Sex	Female	83 (41.9%)	115 (58.1%)	7.866 ***(p=0.005)***
	Male	374 (53.2%)	329 (46.8%)	
	Missing	2	2	
Marital status	Married	218 (48.9%)	228 (51.1%)	4.450 (p=0.349)
	Minor	61 (55.5%)	49 (44.5%)	
	Separated & divorced	20 (40.8%)	29 (59.2%)	
	Single	147 (53.5%)	128 (46.5%)	
	Widow	13 (54.2%)	11 (45.8%)	
	Missing		1	
Religion	Atheist	2 (66.7%)	1 (33.3%)	2.113 (p=0.609)
	Christian	433 (50.1%)	432 (49.9%)	
	Hindu	1 (33.3%)	2 (66.7%)	
	Muslim	15 (62.5%)	9 (37.5%)	
	Missing	8	2	
Occupation	Businessman/woman	60 (52.6%)	54 (47.4%)	1.416 (p=0.841)
	Casual	204 (50.6%)	199 (49.4%)	
	Employed	64 (47.4%)	71 (52.6%)	
	Other	33 (55.9%)	26 (44.1%)	
	unemployed	92 (50.3%)	91 (49.7%)	
	Missing	6	5	
Education	None	33 (60.0%)	22 (40.0%)	***0.001***
	Pre-school	18 (81.8%)	4 (18.2%)	
	Primary	157 (51.0%)	151 (49.0%)	
	Secondary	166 (52.4%)	151 (47.6%)	
	Tertiary	74 (40.7%)	108 (59.3%)	
	Missing	11	10	
Smoking	No	348 (51.2%)	332 (48.8%)	0.078 (p=0.780)
	Yes	89 (50.0%)	89 (50.0%)	
	Missing	22	25	
Alcohol	No	258 (50.1%)	257 (49.9%)	0.410 (p=0.522)
	Yes	180 (52.3%)	164 (47.7%)	
	Missing	21	25	

Legend: The table shows the independent categorical variables abstracted from the patient charts before and after enforcement of referral guidelines. Missing values ranged from 1 to 25 and were excluded from the analysis. Statistical analysis was determined by Pearson χ^2 statistics at 95% CI using SPSS version 21.0.

Effect of enforcement of the referral guidelines on orthopaedic and trauma admissions at KNH

The proportion of walk-ins declined from 248 (54.9%) to 204 (45.1%) after the enforcement of the referral guidelines, while facility referrals increased from 211 (46.6%) to 242 (53.4%) during the same period (p=0.013) (Table 2). After controlling for possible confounders (sex and education level), the odds of walk-ins were 1.653 (95% CI: 1.244

– 2.197) as compared to facility referrals before the enforcement of referral guidelines This means the walk-ins were significantly high before the enforcement of the referral guidelines and this reduced after the enforcement of the referral guidelines during the study period.

Table 2: Walk-ins and Facility Referrals before and after enforcement of referral guidelines, 2021

	Before (n=459)	After (n=446)	Chi-square; p-value	Logistic regression
Walk-ins	248 (54.9%)	204 (45.1%)	***6.219; p=0.013***	***1.653 (95% CI: 1.244 – 2.197) (p=0.001)***
Facility referrals	211 (46.6%)	242 (53.4%)		

Legend: Pearson χ^2 statistics test was used to test for statistical significance at 95% CI and Odds Ratio Estimate was used to test the strength of association.

Although the majority of admissions were admitted through Accident and Emergency (A&E) department, there was a notable decline from 386 (84.1%) and 321 (72.0%) after enforcement of the referral guidelines (p=0.0001) with the least admissions through the Orthopaedic Clinics (OC). There was also a notable increase in admissions through Corporate Outpatient Care (COC) after enforcement of the referral guidelines from 48 (10.5%) to 87 (19.5%) (p=0.011) (Table 3). Overall, there was a significant decline in admissions through A&E and an increase in admissions thorough the COC and OC after the enforcement of the referral guidelines.

Table 3: Orthopaedic and trauma admissions to KNH stratified by point of admission, 2021.

	Before	After	Total	p-value
Accident & Emergency (A&E)	386 (84.1%)	321 (72.0%)	707 (78.1%)	***0.0001***
Corporate Outpatient Clinic (COC)	48 (10.5%)	87 (19.5%)	135 (14.9%)	***0.011***
Orthopaedic Clinics (OC)	25 (5.4%)	38 (8.5%)	63 (7.0%)	0.315
Total	459 (100.0%)	446 (100.0%)	905 (100.0%)	

Legend: Pearson χ^2 statistics test was used to test for statistical significance at 95% CI and Odds Ratio Estimate was used to test the strength of association.

The type of admissions was also reviewed before and after enforcement of referral guidelines. Emergency admissions declined from about 387 (84.7%) to 325 (73.4%), while elective cases increased from 70 (15.3%) to 118 (26.6%) %) (0.0001) after enforcement of the referral guidelines (Table 4). Elective admissions were 50.2% less likely to occur before as opposed to after enforcement of referral guidelines (Table 4). This means that elective admissions increased while emergency admissions reduced after the enforcement of the referral guidelines.

Table 4: Type of orthopaedic and trauma admissions before and after enforcement of referral guidelines, 2021

Characteristics		Before	After	Chi-square test; p-value	OR Estimate (95% CI)
Type of admission	Elective	70 (15.3%)	118 (26.6%)	***17.441; p=0.0001***	***0.498 (0.358 – 0.693)***
	Emergency	387 (84.7%)	325 (73.4%)		
	Total	457 (100.0%)	443 (100.0%)		

Legend: Pearson χ^2 statistics test was used to test for statistical significance at 95% CI and Odds Ratio was used to test the strength of association.

According to the Kenya Health Sector Referral Implementation Guidelines, 2014, facility referrals are expected to be accompanied by official written referral letters from the referring facilities to KNH. The study sought to determine the enforcement of this referral requirement. The study revealed the proportion of referrals with official written referral letters remained unchanged before and after enforcement of the referral guidelines (p=0.860) (Table 5). This means that the enforcement of the referral guidelines had no statistically significant effect on the proportion of admissions with written referral letters as required by the referral guidelines.

Table 5: Facility referrals with referral letters before and after enforcement of referral guidelines, 2021

		Facility referrals		Chi-square; p-value
		Before	After	
Have referral letters	No	109 (51.7%)	123 (50.8%)	0.031; p=0.860
	Yes	102 (48.3%)	119 (49.2%)	
Total		211 (100.0%)	242 (100.0%)	

Legend: Pearson χ^2 statistics test was used to test for statistical significance at 95% CI.

Binary Logistic regression was used to estimate the probability of occurrence of key characteristics before and after the enforcement of the referral guidelines, namely, sex, education level, and mode of payment. The study revealed that the likelihood of being male was 1.575 (1.145 – 2.166) more compared to females before the enforcement of the referral guidelines compared to those after the enforcement of the referral guidelines (0.005) (Table 6). This means that there were fewer male admissions after enforcement of the referral guidelines.

With regard to education level, the study demonstrated that the likelihood of having a tertiary level of education was about 54.4% less before the enforcement of the referral guidelines as opposed to after the enforcement of the referral guidelines (p=0.001) (Table 6). This means that more orthopedic and trauma admissions had a tertiary level of education after enforcement of the referral guidelines.

Regarding the mode of payment, the odds of being a cash payer were 1.846 (1.387 – 2.458) more before compared to after the enforcement of referral guidelines while the likelihood of having insurance cover was 46.2% less likely before enforcement of referral guidelines compared to after enforcement of referral guidelines (Table 6). This means that the enforcement of the referral guidelines was associated with a statistically

significant increase in the number of orthopedic and trauma admissions with active insurance cover.

Table 6: Multivariable analysis of socio-demographic characteristics of orthopaedic admissions to KNH before and after enforcement of referral guidelines, 2021

Characteristics		Before	After	Chi-square test; p-value	Logistic regression (95% CI)
Sex	Female	83 (18.2%)	115 (25.9%)	7.866; p=0.005	1.0 (REF)
	Male	374 (81.8%)	329 (74.1%)		***1.575 (1.145 – 2.166)***
	Total	457 (50.7%)	444 (49.3%)		
Education level	None	33 (7.4%)	22 (5.0%)	18.128; p=0.001	1.0(REF)
	Pre-school	18 (4.0%)	4 (0.9%)		3.0 (0.894 – 10.063)
	Primary	157 (35.0%)	151(34.6%)		0.693 (0.387 - 1.243)
	Secondary	166 (37.1%)	151 (34.6%)		0.733 (0.409 – 1.313)
	Tertiary	74 (16.5%)	108 (24.8%)		***0.457 (0.247 – 0.845)***
	Total	448 (50.7%)	436 (49.3%)		
Mode of payment	Cash	344 (75.3%)	277 (62.2%)	17.837; p=0.0001	1.0(REF)
	Insurance	113 (24.7%)	168 (37.8%)		***0.538 (0.404 – 0.716)***
	Total	457 (50.7%)	445 (49.3%)		

Legend: Pearson χ^2 statistics test was used to test for statistical significance at 95% CI and Odds Ratio was used to test the strength of association. (REF) refers to reference group.

The mode of payment and its association with sex was reviewed and it showed no statistically significant association before the enforcement of the guidelines. However, males were 2.366 times more likely to be cash payers compared to females after enforcement of the referral guidelines (Table 7). This means more females admissions had insurance cover after enforcement of referral guidelines.

Table 7: Multivariable analysis of sex of orthopaedic admissions to KNH before and after enforcement of referral guidelines, 2021 disaggregated by mode of payment

	Sex	Cash	Insurance	Chi-square, p-value	OR (95% CI)
Before	Female	58 (17.0%)	25 (22.1%)	1.519; p=0.218	1.0 (REF)
	Male	284 (83.0%)	88 (77.9%)		1.391 (0.822 – 2.355)
After	Female	54 (19.6%)	61 (36.5%)	***15.574; p=0.0001***	1.0 (REF)
	Male	222 (80.4%)	106 (63.5%)		***2.366 (1.534 – 3.649)***

Legend: Pearson χ^2 statistics test was used to test for statistical significance at 95% CI and Odds Ratio was used to test the strength of association. (REF) refers to reference group.

The study revealed that amongst walk-ins, orthopaedic and trauma admissions, those aged 15 – 24 years and 25 – 64 years had statistically significant decline in admissions through the A&E after enforcement of the guidelines (Table 8). This means the

enforcement of the referral guidelines were associated with less admissions in through A&E and more admissions through the OC and COC.

Table 8: Association between age and point of admission before and after enforcement of referral guidelines stratified by Facility referral and Walk-ins, 2021

	Age category	Point of admission	Before	After	Chi-square, p-value
Walk-ins	0 – 14 years	A&E	13 (72.2%)	5 (27.8%)	1.210; p=0.546
		Clinic	6 (54.5%)	5 (45.5%)	
		COC	5 (55.6%)	4 (44.4%)	
	15 – 24 years	A&E	49 (69.0%)	22 (31.0%)	***17.811; p=0.0001***
		Clinic	0 (0.0%)	6 (100.0%)	
		COC	2 (20.0%)	8 (80.0%)	
	25 – 64 years	A&E	129 (62.3%)	78 (37.7%)	***13.641; p=0.001***
		Clinic	11 (39.3%)	17 (60.7%)	
		COC	28 (40.0%)	42 (60.0%)	
	Above 65 years	A&E	3 (30.0%)	7 (70.0%)	0.932; p=0.628
		Clinic	0 (0.0%)	2 (100.0%)	
		COC	2 (20.0%)	8 (80.0%)	
Facility referral	0 – 14 years	A&E	30 (55.6%)	24 (44.4%)	0.687; p=0.709
		Clinic	3 (75.0%)	1 (25.0%)	
		COC	2 (66.7%)	1 (33.3%)	
	15 – 24 years	A&E	31 (40.8%)	45 (59.2%)	2.833; p=0.243
		Clinic	1 (100.0%)	0 (0.0%)	
		COC	0 (0.0%)	2 (100.0%)	
	25 – 64 years	A&E	123 (48.2%)	132 (51.8%)	5.169; p=0.075
		Clinic	4 (40.0%)	6 (60.0%)	
		COC	8 (26.7%)	22 (73.3%)	
	Above 65 years	A&E	8 (50.0%)	8 (50.0%)	2.00; p=0.368
		Clinic	0 (0.0%)	1 (100.0%)	
		COC	1 (100.0%)	0 (0.0%)	

Legend: Pearson χ^2 statistics test was used to test for statistical significance at 95% CI.

The study revealed a statistically significant increase in the proportion of non-trauma-related orthopaedic admissions after enforcement of the referral guidelines amongst walk-in admissions (p < 0.001). The odds of trauma-related admissions were 2.829 times higher before enforcement of referral guidelines compared to after enforcement of referral guidelines amongst walk-ins' admissions (Table 9). This means that there was a

statistically significant increase in non-trauma-related admissions after enforcement of the referral guidelines amongst walk-ins.

Table 9: Association between the nature of injury before and after enforcement of referral guidelines, 2021.

	Nature of Injury				
		Before	After	Chi-square, p-value	OR
Walk-in	Trauma related	204 (62.0%)	125 (38.0%)	***23.384; p <0.001***	***2.829 (1.842 – 4.345)***
	Non – Trauma related	45 (36.6%)	78 (63.4%)		
Facility Referral	Trauma related	200 (47.6%)	220 (52.4%)	2.095; p=0.148	1.736 (0.816 – 3.690)
	Non – Trauma related	11 (34.4%)	21 (65.6%)		

Legend: Pearson χ^2 statistics test was used to test for statistical significance at 95% CI and Odds Ratio was used to test the strength of association.

Discussions

Sociodemographic characteristics of the sample population

The study demonstrated over three-quarter of orthopaedic and trauma admissions were male before and after enforcement of referral regulations during the study period. This compares favourably with studies done in Addis Ababa, India, Nepal, Middle East, and Botswana showed male predominates orthopaedic admissions (15-22). In addition, male orthopaedic admissions predominated and peaked at 25 – 64 years, and these admissions declined steadily to 65years of age and orthopaedic admissions were comparable across sex for those above 65 years of age. This compares with a retrospective study done in a tertiary hospital in Nepal that showed similar admission rates from 60 years of age (17).

Based on age groups, children and those above 65 years represented the least orthopaedic admissions during the study period before and after enforcement of the referral guidelines, while the majority of admissions were observed among the age group 25 – 64 years. This compares with a retrospective study on orthopaedic admissions done in Warangal, India, that showed the average age was 41.14 years. There was a bimodal distribution with high rates of admission for young adults up to the age of 35 years old as well as for those above 45 years old (16). Similar studies also depicted low paediatric orthopaedic admissions (23). However, this contrasts with a study done in PCEA Kikuyu Mission Hospital, Kenya, that showed 18.84% orthopaedic admissions were of paediatric age group (22). This could be due to the fact that PCEA Kikuyu Mission Hospital is a private mission hospital and is an established and highly regarded orthopaedic centre in the country.

However, the study revealed an overall increase in female orthopaedic admissions compared to males after the enforcement of referral guidelines. This can be attributed to reduction in the number of emergency admissions through Accident and Emergency department and the reduced number of admissions resulting from the road traffic accident due to the enforcement of the referral guidelines since the majority of these emergency admissions are male.

The enforcement of the referral guidelines was associated with more orthopedic and trauma admissions having a tertiary level of education, non-trauma related conditions and

admissions with active insurance cover. These were driven mainly by admissions through the COC following the enforcement of the referral guidelines.

Effect of the referral guidelines on orthopaedic and trauma admissions at KNH

On 1st July 2021, KNH enforced referral guidelines that were meant to streamline the referral process from peripheral health facilities and allow KNH to manage complex orthopaedic and trauma cases that cannot be handled at lower-level health facilities as per the Kenya Health Sector Referral Implementation Guidelines, 2014. This resulted in a statistically significant reduction of the proportion of walk-ins and an increase in proportion of referral cases. Patients were encouraged to seek services from lower-level health facilities and that they should come to KNH with a written formal referral letter from a lower-level health facility.

There was a significant decline in admissions through A&E and an increase in admissions thorough the COC and OC after the enforcement of the referral guidelines. This means the enforcement of the referral guidelines were associated with more admissions through COC and OC and less admissions through the A&E. And this is in tandem with notable reductions in the proportions of emergency admissions from 84.7% to 73.4% and a relative increase in the proportions of elective admissions. The enforcement of the referral guidelines was associated with more elective orthopaedic and trauma admissions and less emergency orthopaedic and trauma admissions, especially amongst walk-ins aged 15 – 64 years. Majority of these emergency admissions through A&E are young and middle-aged admissions which reported a significant decline after the enforcement of the

referral guidelines. The decline in emergency admissions reflect the fact that these were now being handled at the lower-level facilities as opposed to being referred to KNH.

In addition, there was a statistically significant increase in non-trauma orthopaedic admissions doubling from 12.0% to 22.4% after enforcement of the referral guidelines, and this was a reinforcing the fact that elective orthopaedic admissions increased while emergency orthopaedic admissions reduced after the enforcement of the referral guidelines.

The enforcement of referral guidelines was associated with a statistically significant increase in the proportion of orthopaedic and trauma admissions with active insurance cover for those aged between 15 – 64 years amongst walk-ins' admissions. The increase in the proportion with insurance cover after the enforcement of the referral guidelines could be due to an increase in the number and proportion of female orthopedic admissions through the OC and COC. The increase in the elective cases were driven mostly by elderly women with degenerative musculoskeletal disorders who had tertiary level of education and had active insurance cover. These were admitted mostly through the COC. This is reinforcing the finding that the proportion of non-trauma related admissions increased after enforcement of the referral guidelines.

However, there was no significant difference when it came to facility referrals being accompanied by official written referral letters from the referring facilities to KNH. This is because most of the referrals were verbal over the telephone and once a verbal

consensus had been reached, the referring health facilities did not see the need of writing a formal written referral letter.

Conclusions

In conclusion, the enforcement of the referral guidelines significantly reduced the proportion of walk-in patients from 54.9% to 45.1%, while facility referrals increased from 46.6% to 53.4%. There was also a notable increase in orthopaedic and trauma admissions through the OC and COC after enforcement of the referral guidelines. The proportion of emergency admissions declined while that of elective admissions increased after the enforcement of the referral guidelines. The increase in elective cases were mainly driven by the increase in female admissions with active insurance cover, tertiary level of education, non-trauma related conditions and of older age groups. However, the use of official formal written referral letters did not change due to the enforcement of the referral guidelines.

Recommendations

Recommendations to KNH

1. Strict compliance and adherence to the referral guidelines should continue to be enforced to allow KNH function as a premier referral facility and optimize the utilization of the lower-level health facilities;
2. Develop, dissemination and train health workers on the referral guidelines and referral SOPs;
3. Design, disseminate standard referral tools, such referral forms and registers and enforce their routine usage;

4. Perform regular needs assessments for specialized services so as to be appraised of the actual capacity and limitations of the referring health facilities to facilitate smooth continuous of care from the lower-level facilities to KNH;
5. Provide technical assistance and build capacity to strengthen the referral system at the county level to sustain the gains made.

Recommendations to County Government

1. Ensure the availability of standard referral tools, such as referral forms, registers and other relevant forms for referrals at the facility level;
2. Ensure continuous supportive supervision and capacity building of county facilities in the referral system;
3. Ensure availability of accessible and high-quality health services in the county to reduce unnecessary referrals and allow timely and quality service provision.

Recommendations to Health facilities

1. Ensure the availability and utilization of standard referral tools, such as referral forms, registers and other relevant forms for referrals at the facility level.

Acknowledgements

We would like to sincerely acknowledge the work of Brian Okinyi and Micah J. Kipkemei for commitment and assistance in data collection process.

References

1. Hanche-Olsen TP, Alemu L, Viste A, Wisborg T, Hansen KS. Trauma care in Africa: a status report from Botswana, guided by the World Health Organization's "Guidelines for Essential Trauma Care". World J Surg. 2012;36(10):2371-83.
2. Murray CJL, AD L. The Global Burden of Disease: A Comprehensive Assessment of Mortality and Disability From Diseases Injuries, and Risk Factors in 1990 and Projected to 20201996.
3. Murray CJL, AD L. Global Health Statistics: A Compendium of Incidence Prevalence and Mortality Estimates for Over 200 Conditions1996.
4. Hensher M, Price M, Adomakoh S. Referral Hospitals. 2006.
5. Holdsworth G, Garner PA, Harphan T. Crowded outpatient departments in city hospitals of developing countries: a case study from Lesotho. Int J Health Plann Manage. 1993;8(4):315-24.
6. London L, Bachmann OM. Paediatric utilization of a teaching hospital and a community health center. Predictors of level of care used by children from Khayelitsha, Cape Town. S Afr Med J. 1997;87(1):31-6.
7. Ohara K, Melendez V, Uehara N, Ohi G. Study of a patient referral system in the Republic of Honduras. Health Policy Plan. 1998;13(4):433-45.
8. Sanders D, Kravitz J, Lewin S, McKee M. Zimbabwe's hospital referral system: does it work? Health Policy Plan. 1998;13(4):359-70.
9. Stefanini A. District hospitals and strengthening referral systems in developing countries. World Hosp Health Serv. 1994;30(2):14-9.

10. GoK. Article 6 of the Constitution of Kenya 2010. Nairobi, Kenya2010.

11. MoH. Kenya Health Policy 2014 – 2030. Nairobi, Kenya2014.

12. MoH. https://knh.or.ke/index.php/history/ Nairobi, Kenya2021 [

13. KNH IN-PATIENT STATISTICS REPORT 2018, 2019 & 2020 [Internet]. 2020 [cited June 2020].

14. Casagrande PaS. Biometrics 1978; 34:483-6

15. Ahmed E, Chaka T. The pattern of orthopedic admissions in Tikur Anbessa University Hospital, Addis Ababa. Ethiop Med J. 2005;43(2):85-91.

16. Saikiran Velpula, Laxmi Prasanna Gummadi, Nagaraju Vallepu, Bharath Kumar Dasari, Anchuri. SS. Epidemiology of orthopedic trauma admissions in a multispecialty hospital in Warangal-A retrospective study. Clinical Practice. 2019;16(6).

17. Mishra BN, Jha A, Gupta. Epidemiology of Orthopedic Admissions at A Teaching Hospital of Eastern Nepal. Journal of Nobel Medical College. 2017;6(1):56–62.

18. Suraj Bidary, Suresh Pandey, Hemant Kumar Gupta, Roshani Aryal, Bhattarai K. Pattern of Injury among Orthopedic Inpatients in Teaching Hospital in Nepal. Journal of College of Medical Sciences-Nepal. Oct-Dec 2020;16(4).

19. Chandrashekara CM, George MA, Al-Marboi BSK. Demography of orthopedic admissions in a secondary care hospital in oman. Brunei International Medical Journal 2013;9(4):236-42.

20. Premkumar A, Massawe HH, Mshabaha DJ, R.Foran J, XiaohanYing, Sheth NP. The burden of orthopedic disease presenting to a referral hospital in northern Tanzania. Global Surgery. 2015;2(1):70-5.

21. Manwana ME, Mokone GG, Kebaetse M, T Y. Epidemiology of traumatic orthopedic injuries at Princess Marina Hospital, Botswana. South African Orthopedic Journal. 2018;17(1):41-6.

22. Kihuba E. Epidemiology and medical costs of orthopedic conditions in a tertiary hospital in Kenya; A five-year analysis of admission data. BMJ Yale. 2022.

23. Pan RH, Chang NT, Chu D, Hsu KF, Hsu YN, Hsu JC, et al. Epidemiology of orthopedic fractures and other injuries among inpatients admitted due to traffic accidents: a 10-year nationwide survey in Taiwan. Scientific World Journal. 2014; 2014:637872.

Figure 1 Click here to access/download;Figure;Fig 1.docx

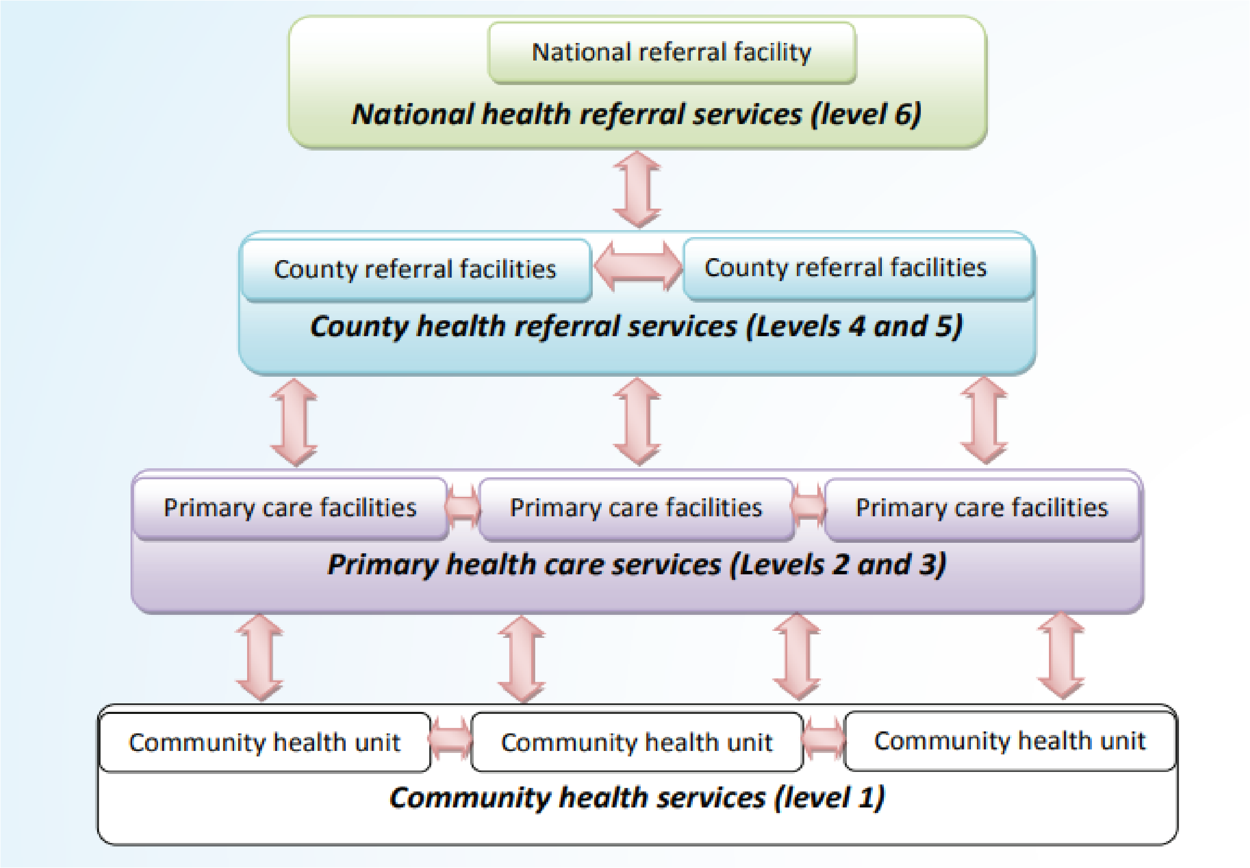

Figure 1: referral linkages between different levels of care (Reproduced from Kenya Health Sector Implementation Referral Guidelines, 2014)

Click here to access/download
Supporting Information
WORKING. 05.06.2022.sav
