# Supplementary material for: Effect of enforcement of the national referral guidelines on patterns of orthopedic admissions to Kenyatta National Hospital, Kenya: Pre-post intervention study
Source: PLoS One. 2024 Aug 13;19(8):e0290195. doi: 10.1371/journal.pone.0290195 (PMC11321550; doi:10.1371/journal.pone.0290195)
Supplement: Attachment — Submitted filename: PONE-D-23-24470_R1.pdf [file pone.0290195.s005.pdf]

PLOS ONE

Effect of enforcement of referral guidelines on patterns of orthopedic and trauma admissions to Kenyatta National Hospital, Kenya: pre-posttest study design

--Manuscript Draft--

**Manuscript Number:**	PONE-D-23-24470R1
**Article Type:**	Research Article
**Full Title:**	Effect of enforcement of referral guidelines on patterns of orthopedic and trauma admissions to Kenyatta National Hospital, Kenya: pre-posttest study design
**Short Title:**	effect of referral guidelines on orthopedic and trauma admissions to Kenyatta National Hospital
**Corresponding Author:**	Maxwell Philip Omondi, PhD<br>University of Nairobi College of Health Sciences: University of Nairobi Faculty of Health Sciences<br>Nairobi, KENYA
**Keywords:**	orthopedic and trauma admissions, Kenyatta National Hospital, referral guidelines
**Abstract:**	Inappropriate utilization of higher-level health facilities and ineffective management of referral processes in resource-limited settings is increasingly becoming a concern in health care management in developing countries. This is characterized by self-referral and frequent bypassing of the nearest health facilities coupled with low formal referral mechanisms. This scenario lends itself to a situation where uncomplicated medical conditions are unnecessarily managed in a high-cost health facility. On July 1, 2021, Kenyatta National Hospital (KNH) did enforce the Kenya Health Sector Referral Implementation Guidelines, 2014 which required patients get approval from KNH referral office and a formal referral letter to be admitted at KNH to reduce the number of walk-ins and allow KNH to function as a referral facility as envisioned by Kenya 2010 Constitution and KNH legal statue of 1987. The study objective was to determine the effect of enforcement of referral guidelines on patterns of orthopaedic and trauma admissions to KNH. This was a pre-post-test study design. Data abstraction was done for 459 and 446 charts before and after enforcement of referral guidelines. Enforcement of the referral guidelines reduced the proportion of walk-in admissions from 54.9% to 45.1%, while proportion of facility referrals increased from 46.6% to 53.4% (p=0.013). The Non-trauma orthopaedic admissions doubled from 12.0% to 22.4% (p<0.001). There was also a notable increase in admissions through the Outpatient Clinic (OC) and Corporate Outpatient Clinic (COC). The proportion of emergency admissions declined while that of elective admissions increased. The increase in elective cases were mainly driven by the increase in female admissions with active insurance cover, tertiary education, non-trauma related conditions and of older age groups. However, use of official formal written referral letters did not change despite the enforcement of referral guidelines. In conclusion, the enforcement of the referral guidelines reduced the proportion of walk-ins' admissions to KNH. The admissions were more non-trauma conditions, had tertiary education level, and more active insurance cover as a result of enforcement of the referral guidelines.
**Order of Authors:**	Maxwell Philip Omondi, PhD
	Fred Chuma Sitati, MD Orthopedic Surgery
**Response to Reviewers:**	We have addressed the reviewers comments to the best of our abilities.<br><br>There is paucity of published literature on this topic and so not much really exist for robust comparison and contrasts in the discussion section
**Additional Information:**	
**Question**	**Response**
**Financial Disclosure**<br><br>Enter a financial disclosure statement that	This study was partially funded by Kenyatta National Hospital (https://knh.or.ke/). MPO received the funding award. The research grant award was RFA 2020/21. The funders had no role in study design, data collection and analysis, decision to publish, or

describes the sources of funding for the work included in this submission. Review the submission guidelines for detailed requirements. View published research articles from *PLOS ONE* for specific examples.<br><br>This statement is required for submission and **will appear in the published article** if the submission is accepted. Please make sure it is accurate.<br><br>**Unfunded studies**<br>Enter: *The author(s) received no specific funding for this work.*<br><br>**Funded studies**<br>Enter a statement with the following details:<br>• Initials of the authors who received each award<br>• Grant numbers awarded to each author<br>• The full name of each funder<br>• URL of each funder website<br>• Did the sponsors or funders play any role in the study design, data collection and analysis, decision to publish, or preparation of the manuscript?<br>• **NO** - Include this sentence at the end of your statement: *The funders had no role in study design, data collection and analysis, decision to publish, or preparation of the manuscript.*<br>• **YES** - Specify the role(s) played.<br><br>* typeset	preparation of the manuscript.
**Competing Interests**<br><br>Use the instructions below to enter a competing interest statement for this submission. On behalf of all authors, disclose any competing interests that could be perceived to bias this work—acknowledging all financial support and any other relevant financial or non-financial competing interests.<br><br>This statement is required for submission and **will appear in the published article** if the submission is accepted. Please make sure it is accurate and that any funding	The authors have declared that no competing interests exist.

sources listed in your Funding Information later in the submission form are also declared in your Financial Disclosure statement.<br><br>View published research articles from *PLOS ONE* for specific examples.<br><br>**NO authors have competing interests**<br><br>Enter: *The authors have declared that no competing interests exist.*<br><br>**Authors with competing interests**<br><br>Enter competing interest details beginning with this statement:<br><br>*I have read the journal's policy and the authors of this manuscript have the following competing interests: [insert competing interests here]*<br><br>* typeset	
**Ethics Statement**<br><br>Enter an ethics statement for this submission. This statement is required if the study involved:<br><br>• Human participants<br>• Human specimens or tissue<br>• Vertebrate animals or cephalopods<br>• Vertebrate embryos or tissues<br>• Field research<br><br>Write "N/A" if the submission does not require an ethics statement.<br><br>General guidance is provided below. Consult the submission guidelines for detailed instructions. **Make sure that all information entered here is included in the Methods section of the manuscript.**	N/A

**Format for specific study types**<br><br>**Human Subject Research (involving human participants and/or tissue)**<br>• Give the name of the institutional review board or ethics committee that approved the study<br>• Include the approval number and/or a statement indicating approval of this research<br>• Indicate the form of consent obtained (written/oral) or the reason that consent was not obtained (e.g. the data were analyzed anonymously)<br><br>**Animal Research (involving vertebrate animals, embryos or tissues)**<br>• Provide the name of the Institutional Animal Care and Use Committee (IACUC) or other relevant ethics board that reviewed the study protocol, and indicate whether they approved this research or granted a formal waiver of ethical approval<br>• Include an approval number if one was obtained<br>• If the study involved *non-human primates*, add *additional details* about animal welfare and steps taken to ameliorate suffering<br>• If anesthesia, euthanasia, or any kind of animal sacrifice is part of the study, include briefly which substances and/or methods were applied<br><br>**Field Research**<br><br>Include the following details if this study involves the collection of plant, animal, or other materials from a natural setting:<br>• Field permit number<br>• Name of the institution or relevant body that granted permission	
**Data Availability**<br><br>Authors are required to make all data underlying the findings described fully available, without restriction, and from the time of publication. PLOS allows rare exceptions to address legal and ethical concerns. See the PLOS Data Policy and FAQ for detailed information.	Yes - all data are fully available without restriction

A Data Availability Statement describing where the data can be found is required at submission. Your answers to this question constitute the Data Availability Statement and **will be published in the article**, if accepted.<br><br>**Important:** Stating 'data available on request from the author' is not sufficient. If your data are only available upon request, select 'No' for the first question and explain your exceptional situation in the text box.<br><br>Do the authors confirm that all data underlying the findings described in their manuscript are fully available without restriction?	
**Describe where the data may be found in full sentences. If you are copying our sample text, replace any instances of XXX with the appropriate details.**<br><br>• If the data are **held or will be held in a public repository**, include URLs, accession numbers or DOIs. If this information will only be available after acceptance, indicate this by ticking the box below. For example: *All XXX files are available from the XXX database (accession number(s) XXX, XXX.*).<br>• If the data are all contained **within the manuscript and/or Supporting Information files**, enter the following: *All relevant data are within the manuscript and its Supporting Information files.*<br>• If neither of these applies but you are able to provide **details of access elsewhere**, with or without limitations, please do so. For example:<br><br>*Data cannot be shared publicly because of [XXX]. Data are available from the XXX Institutional Data Access / Ethics Committee (contact via XXX) for researchers who meet the criteria for access to confidential data.*<br><br>*The data underlying the results presented in the study are available from (include the name of the third party*	All relevant data are within the manuscript and its Supporting Information files.

and contact information or URL).<br>• This text is appropriate if the data are owned by a third party and authors do not have permission to share the data.<br><br>* typeset	
Additional data availability information:	

Manuscript
Click here to access/download;Manuscript;manuscript _ Clean copy.docx

Effect of enforcement of referral guidelines on patterns of orthopedic and trauma admissions to Kenyatta National Hospital, Kenya: pre-posttest study design

Authors: Maxwell Philip Omondi[1¶], Fred Chuma Sitati[1¶]

[1] Orthopedics Unit, Department of Surgery, University of Nairobi, Nairobi, Kenya

*Corresponding author: Maxwell Philip Omondi
Email: maxwellomondi@gmail.com

¶All authors contributed equally to this work.

Abstract

Inappropriate utilization of higher-level health facilities and ineffective management of referral processes in resource-limited settings is increasingly becoming a concern in health care management in developing countries. This is characterized by self-referral and frequent bypassing of the nearest health facilities coupled with low formal referral mechanisms. This scenario lends itself to a situation where uncomplicated medical conditions are unnecessarily managed in a high-cost health facility. On July 1, 2021, Kenyatta National Hospital (KNH) did enforce the Kenya Health Sector Referral Implementation Guidelines, 2014 which required patients get approval from KNH referral office and a formal referral letter to be admitted at KNH to reduce the number of walk-ins and allow KNH to function as a referral facility as envisioned by Kenya 2010 Constitution

and KNH legal statue of 1987. The study objective was to determine the effect of enforcement of referral guidelines on patterns of orthopaedic and trauma admissions to KNH. This was a pre-post-test study design. Data abstraction was done for 459 and 446 charts before and after enforcement of referral guidelines. Enforcement of the referral guidelines reduced the proportion of walk-in admissions from 54.9% to 45.1%, while proportion of facility referrals increased from 46.6% to 53.4% (p=0.013). The Non-trauma orthopaedic admissions doubled from 12.0% to 22.4% (p<0.001). There was also a notable increase in admissions through the Outpatient Clinic (OC) and Corporate Outpatient Clinic (COC). The proportion of emergency admissions declined while that of elective admissions increased. The increase in elective cases were mainly driven by the increase in female admissions with active insurance cover, tertiary education, non-trauma related conditions and of older age groups. However, use of official formal written referral letters did not change despite the enforcement of referral guidelines. In conclusion, the enforcement of the referral guidelines reduced the proportion of walk-ins' admissions to KNH. The admissions had more non-trauma conditions, had tertiary education level, and more active insurance cover as a result of enforcement of the referral guidelines.

Keys: referral guidelines, Orthopaedic and trauma, pattern of admission

Background

Kenya is administratively organized into forty-seven (47) counties with Nairobi Capital city being in Nairobi County (1). Health care service delivery has been devolved to the county level with the national level being responsible for health policy formulation and strategic planning. Kenyatta National Hospital (KNH) falls under national government while County and Subcounty level hospitals fall under County government administratively (1) (2). The Kenya health service delivery system is organized across six levels of care, beginning at the community level (level 1) and continuing through primary care services, which include dispensaries (level 2) and health centers (level 3), and county referral health services (level 4 & 5) all the way to the national referral health services (level 6) (See Fig 1) (2). KNH is a level 6 facility and falls under national government as a premier national referral health facility that offers very specialized skills, expertise and services (2).

Figure 1: Referral linkages between different levels of care (Reproduced from Kenya Health Sector Implementation Referral Guidelines, 2014)

KNH was established in 1901 as a National Referral and Teaching Hospital to provide training and medical research in Kenya. KNH became a State Corporation in 1987 and sits at the apex of the health referral system in Kenya (3). According to the KNH Board order of 1987 contained in the Legal Notice No. 109, the functions of KNH were spelled out as a) to receive patients on referral for specialized health care; b) to provide facilities for medical education for the University of Nairobi and other health allied courses; c) to contribute to national health planning (3). This understanding has been reinforced by the

Kenya Health Sector Referral Implementation Guidelines, 2014, and the Constitution of Kenya 2010 which tasks KNH with the responsibility for health policy formulation (1, 2).

Tertiary hospitals in resource-limited countries treat referred patients but in most cases are the first level of care for the vast majority of patients (4). One of the challenges in health care delivery in resource-limited settings is the inappropriate utilization of tertiary health facilities that results in patients' congestion in referral hospitals with simple conditions that can be effectively managed at lower health facilities. The majority of these patients are self-referred, bypassing lower-level health facilities in the process (5-8). This jeopardizes the appropriate delivery of primary, secondary, and tertiary health care.

Tertiary health care is overwhelmed by the large demand for primary health care that should be managed at lower level health facilities and in the process cripples the primary health care system and this effectively ensures that primary health care facilities remain underused and inefficient (9).

Orthopaedic wards in KNH have consistently recorded the highest bed occupancy percent for the last couple of years. In 2018, 2019, and 2020, it recorded bed occupancy percent of 142.2%, 138.2%, and 116.5%, respectively, with the overall KNH bed occupancy percent of 106.2%, 113.4 percent and 91.5% (10). This coupled with inadequate staffing levels results in low nurse-patient ratio of 1:10 that compromises not only the quality of nursing care given to patients but also the ability of KNH to effectively perform its statutory obligations.

At the strategic level, the government's overall strategic plan, the Second Medium-Term Plan (MTP) of Vision 2030, identifies enforcement of referral guidelines as a key strategy to increase the use of services at lower levels of the health care system and reduce self-referral to higher levels of care. Consequently, on 1st July 2021, KNH management made a decision to reinforce the Kenya Health Sector Referral Guidelines 2014 that places KNH at the tip of the health sector referral system. This meant that patients will be seen based on referral letters from other health facilities to reduce the number of walk-in patients who would have otherwise been appropriately seen at the peripheral health facilities. This will contribute to the decongestion of KNH Orthopaedic and trauma admissions. The purpose of this study was to determine the effect of the enforcement of referral guidelines on the patterns of orthopaedic and trauma admissions to Kenyatta National Hospital.

Methods and Materials

Study design: This was a pre-post-test study design. Enforcement of referral guidelines required that the referring health facility consults with KNH referral coordination Office for concurrence before patients are referred to KNH. This was to ensure only patients who require specialized medical and orthopaedic and trauma care not available at the peripheral health facilities get admitted to KNH.

Study area: KNH is the largest teaching and referral hospital in East and Central Africa. KNH Orthopaedic Wards were the study area. KNH is based in Upperhill, Nairobi, the capital city of Kenya. It is located along Hospital Road, about 5km from the city centre. KNH has a bed capacity of 1,800, 6,000+ staff members, 50 wards, 22 out-patient clinics, 24 theaters (16 specialized) and Accident & Emergency Department(3). Of the 1800 bed capacity, 96 beds are allocated to orthopaedic wards. KNH is a 10-floor storied building

complex and the Orthopaedic wards are located on the 6th floor but we also have admissions in private wings in 9th and 10th floor. Orthopaedic patients with other co-morbidities also get admitted to other wards in KNH.

Study population: Orthopaedic in-patient case loads

Study duration: The study duration was from February 1st to December 31st 2021. It covered 5 months before and 5 months after enforcement of the referral guidelines. The referral guidelines were enforced from July 1, 2021. Inclusion criteria was all orthopaedic and trauma admissions to KNH during the study period. 1st February to 31st December 2021. Data was accessed from the patient charts from 1st January to 31st March 2022 during the data collection period.

Sample size calculation and sampling procedure: Using an adjusted formula for calculating sample sizes that compare two binomial distributions (11), a total of 459 files and 446 files were abstracted before and after the enforcement of the referral guidelines. Stratified sampling technique was used with orthopaedic and trauma admissions being stratified by month of admission and Population Proportionate to Size (PPS) was used to determine the sample size per strata. Systematic random sampling was then used to identify the sampled files using the KNH Health Records Register as the sampling frame from each of these three service entry points {Accident and Emergency (A&E), Orthopedic Clinic (OC), and Corporate Outpatient Clinic (COC)}. This ensured the selected sample would be representative of the admissions by month from each of these three orthopedic and trauma admissions entry points. Informed consent, auditory, and visual confidentiality were provided.

Variables: variables abstracted for this study were a) Socio-demographic characteristics – age, sex, marital status, religion, education level b) Nature of admission; c) Occupation; d) Type of admission; e) Mode of payment;

Data collection procedures: Data collection was done through a data abstraction form. This was a manual survey tool. Data were abstracted from the patient charts and transferred to the data abstraction form by the research assistants.

Quality assurance & quality control procedures: Three Research assistants underwent two (2) day training on the study protocol. A pilot study was conducted during the design of the study protocol to test the data collection tools for relevance, appropriateness to answer the research questions, and adjustments of the data collection tools made as necessary. The Principal Investigator conducted daily reviews of all abstracted forms, verified for accuracy, completeness, and compliance with the research protocol. Inaccurate, incomplete and non-compliance entries were repeated under the supervision of the Principal Investigator as part on ongoing training and to ensure similar errors are minimized.

Ethical considerations: UoN/KNH Ethics and Research Committee granted ethical approval (ERC No: P852/10/2021). Administrative approval was also sought from KNH Medical Research Department and KNH Orthopaedics Department. This study was carried out in accordance with UoN/KNH Ethics and Research Committee guidelines and regulations. For the underage, formal verbal consent was obtained from the parent/guardian.

Data management and analysis plan: Data abstraction tool was used to collect quantitative data. The data were entered into a password-protected Redcap database.

Descriptive analysis was done using measures of central tendency (mean), measures of dispersions such as range, standard deviation were used, frequency distribution were also used. Inferential analysis was done using Pearsons's chi-square test, logistic regression, odds ratio to determine effect of the enforcement of the referral guidelines and also determine possible associations. SPSS version 21 was used.

Study limitations: a) Incomplete and missing records – this was addressed by increasing the sample size by 10%; b) Effect of COVID 19 pandemic on referrals of cases from peripheral health facilities and walk-in patients – this was addressed by ensuring the data collection period covered the covid period when the restrictions of intercounty movements were already lifted though emergency referrals were never lifted during the entire COVID period. Despite these limitations, given the paucity of published literature in this filed, this study offers key information on the effects of enforcement of referral guidelines in Orthopaedic and trauma admissions in KNH with important lessons for Kenya and possible sub-Saharan Africa.

Results

I) Socio-demographic characteristics of the sample population

The total number of charts abstracted was 905 with 459 charts before and 446 charts after the enforcement of the referral guidelines.

Key socio-demographic characteristics of orthopaedic and trauma admissions at KNH such as age, sex, education, occupation, marital status, smoking and alcohol status were reviewed and compared between the two groups. Majority of the admissions were aged between 25 – 64 years with children and elderly (above 65 years) represented the least admissions. Over three quarter of the admissions were male before and after the

enforcement of referral guidelines. The male admissions peaked at 25 – 64 years of age and declined steadily to 65years of age and orthopaedic admissions were comparable across sex for those above 65 years of age. A good proportion of the admissions had primary and secondary level of education (Table 1).

Education and sex were noted to be statistically significant between the two groups at p=0.001 and p=0.005, respectively. However, age, religion, occupation, marital status, smoking, and alcohol intake habits were comparable amongst the two groups (P > 0.05) (Table 1).

Table 1: Socio-demographic characteristics between orthopaedic and trauma admissions at KNH before and after enforcement of the referral guidelines, 2021

Variable	Category	Before (n=459)	After (n=446)	Chi-square, p-value
Age	0 – 14 years	59 (59.6%)	40 (40.4%)	7.121 (p=0.068)
	15 – 24 years	83 (50.0%)	83 (50.0%)	
	25 – 64 years	303 (50.5%)	297 (49.5%)	
	Above 65 years	14 (35.0%)	26 (65.0%)	
Sex	Female	83 (41.9%)	115 (58.1%)	7.866 ***(p=0.005)***
	Male	374 (53.2%)	329 (46.8%)	
	Missing	2	2	
Marital status	Married	218 (48.9%)	228 (51.1%)	4.450 (p=0.349)
	Minor	61 (55.5%)	49 (44.5%)	
	Separated & divorced	20 (40.8%)	29 (59.2%)	
	Single	147 (53.5%)	128 (46.5%)	
	Widow	13 (54.2%)	11 (45.8%)	
	Missing		1	
Religion	Atheist	2 (66.7%)	1 (33.3%)	2.113 (p=0.609)
	Christian	433 (50.1%)	432 (49.9%)	
	Hindu	1 (33.3%)	2 (66.7%)	
	Muslim	15 (62.5%)	9 (37.5%)	
	Missing	8	2	
Occupation	Businessman/woman	60 (52.6%)	54 (47.4%)	1.416 (p=0.841)
	Casual	204 (50.6%)	199 (49.4%)	
	Employed	64 (47.4%)	71 (52.6%)	
	Other	33 (55.9%)	26 (44.1%)	
	unemployed	92 (50.3%)	91 (49.7%)	
	Missing	6	5	
Education	None	33 (60.0%)	22 (40.0%)	***0.001***
	Pre-school	18 (81.8%)	4 (18.2%)	
	Primary	157 (51.0%)	151 (49.0%)	
	Secondary	166 (52.4%)	151 (47.6%)	
	Tertiary	74 (40.7%)	108 (59.3%)	
	Missing	11	10	
Smoking	No	348 (51.2%)	332 (48.8%)	0.078 (p=0.780)
	Yes	89 (50.0%)	89 (50.0%)	
	Missing	22	25	
Alcohol	No	258 (50.1%)	257 (49.9%)	0.410 (p=0.522)
	Yes	180 (52.3%)	164 (47.7%)	
	Missing	21	25	

Legend: The table shows the independent categorical variables abstracted from the patient charts before and after enforcement of referral guidelines. Missing values ranged from 1 to 25 and were excluded from the analysis. Statistical analysis was determined by Pearson χ^2 statistics at 95% CI using SPSS version 21.0.

II) Effect of enforcement of the referral guidelines on orthopaedic and trauma admissions at KNH

a) Profile of admissions to KNH

The effect of enforcement of the referral guidelines on profile of admissions namely nature of admissions (walk-ins vs facility referrals), point of admissions (A&E, OC, COC), types of admissions (emergency vs electives) and formal referrals were reviewed.

The proportion of walk-ins declined from 248/459 (54.9%) to 204/446 (45.1%) after the enforcement of the referral guidelines, while facility referrals increased from 211 (46.6%) to 242 (53.4%) during the same period (p=0.013) (Table 2). After controlling for possible confounders (sex and education level), the odds of walk-ins were 1.653 (95% CI: 1.244 – 2.197) as compared to facility referrals before the enforcement of referral guidelines This the walk-ins were significantly high before the enforcement of the referral guidelines and this reduced after the enforcement of the referral guidelines during the study period. This means that enforcement of the referral guidelines did reduce the proportion of admissions that were self-referred with a proportional increase in admissions that were facility referred.

Table 2: Walk-ins and Facility Referrals before and after enforcement of referral guidelines, 2021

	Before (n=459)	After (n=446)	Chi-square; p-value	Logistic regression
Walk-ins	248 (54.9%)	204 (45.1%)	***6.219; p=0.013***	***1.653 (95% CI: 1.244 – 2.197) (p=0.001)***
Facility referrals	211 (46.6%)	242 (53.4%)		

Legend: Pearson χ^2 statistics test was used to test for statistical significance at 95% CI and Odds Ratio Estimate was used to test the strength of association.

Although the majority of admissions were admitted through Accident and Emergency (A&E) department, there was a notable decline from 386 (84.1%) and 321 (72.0%) after enforcement of the referral guidelines (p=0.0001) with the least admissions through the Orthopaedic Clinics (OC). There was also a notable increase in admissions through Corporate Outpatient Care (COC) after enforcement of the referral guidelines from 48 (10.5%) to 87 (19.5%) (p=0.011) (Table 3). Overall, there was a significant decline in admissions through A&E and an increase in admissions thorough the COC and OC after the enforcement of the referral guidelines.

Table 3: Orthopaedic and trauma admissions to KNH stratified by point of admission, 2021.

	Before	After	Total	p-value
Accident & Emergency (A&E)	386 (84.1%)	321 (72.0%)	707 (78.1%)	***0.0001***
Corporate Outpatient Clinic (COC)	48 (10.5%)	87 (19.5%)	135 (14.9%)	***0.011***
Orthopaedic Clinics (OC)	25 (5.4%)	38 (8.5%)	63 (7.0%)	0.315
Total	459 (100.0%)	446 (100.0%)	905 (100.0%)	

Legend: Pearson χ^2 statistics test was used to test for statistical significance at 95% CI and Odds Ratio Estimate was used to test the strength of association.

The type of admissions was also reviewed before and after enforcement of referral guidelines. Emergency admissions declined from about 387 (84.7%) to 325 (73.4%), while elective cases increased from 70 (15.3%) to 118 (26.6%) %) (0.0001) after enforcement of the referral guidelines (Table 4). Elective admissions were 50.2% less likely to occur before as opposed to after enforcement of referral guidelines (Table 4). This means that elective admissions increased while emergency admissions reduced after the enforcement of the referral guidelines.

Table 4: Type of orthopaedic and trauma admissions before and after enforcement of referral guidelines, 2021

Characteristics		Before	After	Chi-square test; p-value	OR Estimate (95% CI)
Type of admission	Elective	70 (15.3%)	118 (26.6%)	***17.441; p=0.0001***	***0.498 (0.358 – 0.693)***
	Emergency	387 (84.7%)	325 (73.4%)		
	Total	457 (100.0%)	443 (100.0%)		

Legend: Pearson χ^2 statistics test was used to test for statistical significance at 95% CI and Odds Ratio was used to test the strength of association.

The study sought to determine the proportion of admissions accompanied by official written referral letters from the referring health facilities to KNH. The study revealed the proportion of referrals with official written referral letters remained unchanged before and after enforcement of the referral guidelines (p=0.860) (Table 5). This means that the enforcement of the referral guidelines had no statistically significant effect on the proportion of admissions with written referral letters as required by the referral guidelines.

Table 5: Facility referrals with referral letters before and after enforcement of referral guidelines, 2021

		Facility referrals		Chi-square; p-value
		Before	After	
Have referral letters	No	109 (51.7%)	123 (50.8%)	0.031; p=0.860
	Yes	102 (48.3%)	119 (49.2%)	
Total		211 (100.0%)	242 (100.0%)	

Legend: Pearson χ^2 statistics test was used to test for statistical significance at 95% CI.

b) Key socio-demographic patterns of admissions to KNH

Binary Logistic regression was used to estimate the probability of occurrence of key socio-demographic patterns of admissions before and after the enforcement of the referral guidelines, namely, sex, education level, and mode of payment. The study revealed that the likelihood of being male was 1.575 (1.145 – 2.166) less likely compared to females after the enforcement of the referral guidelines compared to those after the enforcement

of the referral guidelines (0.005) (Table 6). This means that there were fewer male admissions after enforcement of the referral guidelines.

With regard to education level, the study demonstrated that the likelihood of having a tertiary level of education was about 54.4% more after the enforcement of the referral guidelines as opposed to after the enforcement of the referral guidelines (p=0.001) (Table 6). This means that more orthopedic and trauma admissions had a tertiary level of education after enforcement of the referral guidelines.

Regarding the mode of payment, the odds of being a cash payer were 1.846 (1.387 – 2.458) more before compared to after the enforcement of referral guidelines while the likelihood of having insurance cover was 46.2% less likely before enforcement of referral guidelines compared to after enforcement of referral guidelines (Table 6). This means that the enforcement of the referral guidelines was associated with a statistically significant increase in the number of orthopedic and trauma admissions with active insurance cover.

Table 6: Multivariable analysis of key socio-demographic characteristics of orthopaedic admissions to KNH before and after enforcement of referral guidelines, 2021

Characteristics		Before	After	Chi-square test; p-value	Logistic regression (95% CI)
Sex	Female	83 (18.2%)	115 (25.9%)	7.866; p=0.005	1.0 (REF)
	Male	374 (81.8%)	329 (74.1%)		***1.575 (1.145 – 2.166)***
	Total	457 (50.7%)	444 (49.3%)		
Education level	None	33 (7.4%)	22 (5.0%)	18.128; p=0.001	1.0(REF)
	Pre-school	18 (4.0%)	4 (0.9%)		3.0 (0.894 – 10.063)
	Primary	157 (35.0%)	151(34.6%)		0.693 (0.387 - 1.243)
	Secondary	166 (37.1%)	151 (34.6%)		0.733 (0.409 – 1.313)
	Tertiary	74 (16.5%)	108 (24.8%)		***0.457 (0.247 – 0.845)***
	Total	448 (50.7%)	436 (49.3%)		

Mode of payment	Cash	344 (75.3%)	277 (62.2%)	17.837; p=0.0001	1.0(REF)
	Insurance	113 (24.7%)	168 (37.8%)		***0.538 (0.404 – 0.716)***
	Total	457 (50.7%)	445 (49.3%)		

Legend: Pearson χ^2 statistics test was used to test for statistical significance at 95% CI and Odds Ratio was used to test the strength of association. (REF) refers to reference group.

The mode of payment and its association with sex was reviewed and it showed no statistically significant association before the enforcement of the guidelines. However, males were 2.366 times more likely to be cash payers compared to females after enforcement of the referral guidelines (Table 7). This means more females admissions had insurance cover after enforcement of referral guidelines.

Table 7: Multivariable analysis of sex of orthopaedic admissions to KNH before and after enforcement of referral guidelines, 2021 disaggregated by mode of payment

	Sex	Cash	Insurance	Chi-square, p-value	OR (95% CI)
Before	Female	58 (17.0%)	25 (22.1%)	1.519; p=0.218	1.0 (REF)
	Male	284 (83.0%)	88 (77.9%)		1.391 (0.822 – 2.355)
After	Female	54 (19.6%)	61 (36.5%)	***15.574; p=0.0001***	1.0 (REF)
	Male	222 (80.4%)	106 (63.5%)		***2.366 (1.534 – 3.649)***

Legend: Pearson χ^2 statistics test was used to test for statistical significance at 95% CI and Odds Ratio was used to test the strength of association. (REF) refers to reference group.

c) Association between patients' characteristics and nature of admissions

The effect of enforcement of the referral guidelines on patients characteristics (age, point of admissions and nature of injury) on the nature of admissions (walk-ins vs facility referrals) were reviewed.

The study revealed that amongst walk-ins, orthopedic and trauma admissions, those aged 15 – 24 years and 25 – 64 years had statistically significant decline in admissions through the A&E after enforcement of the guidelines (Table 8). This means the

enforcement of the referral guidelines were associated with less admissions in through A&E and more admissions through the OC and COC.

Table 8: Association between age and point of admission before and after enforcement of referral guidelines stratified by Facility referral and Walk-ins, 2021

	Age category	Point of admission	Before	After	Chi-square, p-value
Walk-ins	0 – 14 years	A&E	13 (72.2%)	5 (27.8%)	1.210; p=0.546
		Clinic	6 (54.5%)	5 (45.5%)	
		COC	5 (55.6%)	4 (44.4%)	
	15 – 24 years	A&E	49 (69.0%)	22 (31.0%)	***17.811; p=0.0001***
		Clinic	0 (0.0%)	6 (100.0%)	
		COC	2 (20.0%)	8 (80.0%)	
	25 – 64 years	A&E	129 (62.3%)	78 (37.7%)	***13.641; p=0.001***
		Clinic	11 (39.3%)	17 (60.7%)	
		COC	28 (40.0%)	42 (60.0%)	
	Above 65 years	A&E	3 (30.0%)	7 (70.0%)	0.932; p=0.628
		Clinic	0 (0.0%)	2 (100.0%)	
		COC	2 (20.0%)	8 (80.0%)	
Facility referral	0 – 14 years	A&E	30 (55.6%)	24 (44.4%)	0.687; p=0.709
		Clinic	3 (75.0%)	1 (25.0%)	
		COC	2 (66.7%)	1 (33.3%)	
	15 – 24 years	A&E	31 (40.8%)	45 (59.2%)	2.833; p=0.243
		Clinic	1 (100.0%)	0 (0.0%)	
		COC	0 (0.0%)	2 (100.0%)	
	25 – 64 years	A&E	123 (48.2%)	132 (51.8%)	5.169; p=0.075
		Clinic	4 (40.0%)	6 (60.0%)	
		COC	8 (26.7%)	22 (73.3%)	
	Above 65 years	A&E	8 (50.0%)	8 (50.0%)	2.00; p=0.368
		Clinic	0 (0.0%)	1 (100.0%)	
		COC	1 (100.0%)	0 (0.0%)	

Legend: Pearson χ^2 statistics test was used to test for statistical significance at 95% CI.

The study revealed a statistically significant increase in the proportion of non-trauma-related orthopaedic admissions after enforcement of the referral guidelines amongst walk-in admissions ($p < 0.001$). The odds of non-trauma-related admissions were 2.829 times higher after enforcement of referral guidelines amongst walk-ins' admissions (Table 9). This means that there was a statistically significant increase in non-trauma-related admissions after enforcement of the referral guidelines amongst walk-ins.

Table 9: Association between the nature of injury before and after enforcement of referral guidelines stratified by Facility referral and Walk-ins, 2021.

	Nature of Injury				
		Before	After	Chi-square, p-value	OR
Walk-in	Trauma related	204 (62.0%)	125 (38.0%)	***23.384; p <0.001***	***2.829 (1.842 – 4.345)***
	Non – Trauma related	45 (36.6%)	78 (63.4%)		
Facility Referral	Trauma related	200 (47.6%)	220 (52.4%)	2.095; p=0.148	1.736 (0.816 – 3.690)
	Non – Trauma related	11 (34.4%)	21 (65.6%)		

Legend: Pearson χ^2 statistics test was used to test for statistical significance at 95% CI and Odds Ratio was used to test the strength of association.

Discussions

I) Sociodemographic characteristics of the sample population

The study demonstrated over three-quarter of orthopaedic and trauma admissions were male before and after enforcement of referral regulations during the study period. This compares favourably with studies done in Addis Ababa, India, Nepal, Middle East, and Botswana showed male predominates orthopaedic admissions (12-19). In addition, male orthopaedic admissions predominated and peaked at 25 – 64 years, and these admissions declined steadily to 65years of age and orthopaedic admissions were comparable across sex for those above 65 years of age. This compares with a retrospective study done in a tertiary hospital in Nepal that showed similar admission rates from 60 years of age (14).

Based on age groups, children and those above 65 years represented the least orthopaedic admissions during the study period before and after enforcement of the referral guidelines, while the majority of admissions were observed among the age group 25 – 64 years. This compares with a retrospective study on orthopaedic admissions done

in Warangal, India, that showed the average age was 41.14 years. There was a bimodal distribution with high rates of admission for young adults up to the age of 35 years old as well as for those above 45 years old (13). Similar studies also depicted low paediatric orthopaedic admissions (20). However, this contrasts with a study done in PCEA Kikuyu Mission Hospital, Kenya, that showed 18.84% orthopaedic admissions were of paediatric age group (19). This could be due to the fact that PCEA Kikuyu Mission Hospital is an established and highly regarded specialized orthopaedic centre in the country.

However, the study revealed an overall increase in female orthopaedic admissions compared to males after the enforcement of referral guidelines. This can be attributed to reduction in the number of emergency admissions through Accident and Emergency department and the reduced number of admissions resulting from the road traffic accident due to the enforcement of the referral guidelines since the majority of these emergency admissions are male.

The enforcement of the referral guidelines was associated with more orthopedic and trauma admissions having a tertiary level of education, non-trauma related conditions and admissions with active insurance cover. These may have been driven mainly by admissions through the COC following the enforcement of the referral guidelines. COC is the private clinic wing of KNH.

II) Effect of the referral guidelines on orthopaedic and trauma admissions at KNH

On 1st July 2021, KNH enforced referral guidelines that were meant to streamline the referral process from peripheral health facilities and allow KNH to a) manage complex

orthopaedic and trauma cases that cannot be handled at lower-level health facilities as per the Kenya Health Sector Referral Implementation Guidelines, 2014 b) perform its statutory functions. This resulted in a statistically significant reduction of walk-ins and an increase in facility referral cases. Patients were encouraged to seek services from lower-level health facilities and that they should come to KNH with a written formal referral letter from a lower-level health facility.

There was a significant decline in admissions through A&E and an increase in admissions through the COC and OC after the enforcement of the referral guidelines. This means the enforcement of the referral guidelines were associated with more admissions through COC and OC and less admissions through the A&E. And this is in tandem with notable reductions in the proportions of emergency admissions from 84.7% to 73.4% and a relative increase in the proportions of elective admissions. The enforcement of the referral guidelines was associated with more elective orthopaedic and trauma admissions and less emergency orthopaedic and trauma admissions, especially amongst walk-ins aged 15 – 64 years. Majority of these emergency admissions through A&E are young and middle-aged admissions which reported a significant decline after the enforcement of the referral guidelines. The decline in emergency admissions reflect the fact that maybe these were now being handled at the lower-level facilities as opposed to being referred to KNH.

In addition, there was a statistically significant increase in non-trauma orthopaedic admissions doubling from 12.0% to 22.4% after enforcement of the referral guidelines, and this was a reinforcing the fact that elective orthopaedic admissions increased while

emergency orthopaedic admissions reduced after the enforcement of the referral guidelines. Non- trauma orthopaedic cases are usually elective admissions.

The enforcement of referral guidelines was associated with a statistically significant increase in the proportion of orthopaedic and trauma admissions with active insurance cover for those aged between 15 – 64 years amongst walk-ins' admissions. The increase in the proportion with insurance cover after the enforcement of the referral guidelines could be due to an increase in the number and proportion of female orthopedic admissions through the OC and COC. The increase in the elective cases were driven mostly by elderly women with degenerative musculoskeletal disorders who had tertiary level of education and had active insurance cover. These were admitted mostly through the COC. This is reinforcing the finding that the proportion of non-trauma related admissions increased after enforcement of the referral guidelines.

However, there was no significant difference when it came to facility referrals being accompanied by official written referral letters from the referring facilities to KNH. This compares with study done in Moi Teaching and Referral Hospital on adherence to paediatric national referral protocol that revealed poor adherence with only 15.9% accounting for hospital referrals with a formal referral letters (21). A study in Uganda also revealed poor adherence to referral guidelines by Community Health Workers referring patients to health facilities (22). The low use of referral letters in the current study could be because most of the referrals were verbal over the telephone and once a verbal consensus had been reached, the referring health facilities did not see the need of writing

a formal written referral letter. However, this contrasts with a study on adherence to referral guidelines and use of referral letters in Cape Town, South Africa and in Malawi that revealed over 90% adherence to referral guidelines (23, 24). This is likely due to the fact that Red Cross War Memorial Children's Hospital in Cape Town, South Africa and Queen Elizabeth Central Hospitals in Malawi were specialized treatment centres.

Conclusions

In conclusion, the enforcement of the referral guidelines significantly reduced the proportion of walk-in patients from 54.9% to 45.1%, while facility referrals increased from 46.6% to 53.4%. There was also a notable increase in orthopaedic and trauma admissions through the OC and COC after enforcement of the referral guidelines. The proportion of emergency admissions declined while that of elective admissions increased after the enforcement of the referral guidelines. The increase in elective cases were mainly driven by the increase in female admissions with active insurance cover, tertiary level of education, non-trauma related conditions and of older age groups. However, the use of official formal written referral letters did not change due to the enforcement of the referral guidelines.

Recommendations

Recommendations to KNH Orthopaedic Department

1. Strict compliance and adherence to the referral guidelines should continue to be enforced to allow KNH function as a premier referral facility and optimize the utilization of the lower-level health facilities;
2. Develop, disseminate and train health workers on the referral guidelines and referral SOPs;

3. Design, disseminate standard referral tools, such referral forms and registers and enforce their routine usage;
4. Perform regular needs assessments for specialized services so as to be appraised of the actual capacity and limitations of the referring health facilities to facilitate smooth continuum of care from the lower-level facilities to KNH;
5. Provide technical assistance and build capacity to strengthen the referral system at the county level to sustain the gains made.

Recommendations to County Health Management Team

1. Ensure the availability of standard referral tools, such as referral forms, registers and other relevant forms for referrals at the facility level;
2. Ensure continuous supportive supervision and capacity building of county facilities in the referral system;
3. Ensure availability of accessible and high-quality health services in the county to reduce unnecessary referrals and allow timely and quality service provision.

Recommendations to Health facilities

1. Ensure the availability and utilization of standard referral tools, such as referral forms, registers and other relevant forms for referrals at the facility level.

Acknowledgements

We would like to sincerely acknowledge the work of Brian Okinyi and Micah J. Kipkemei for commitment and assistance in data collection process.

References

1. GoK. Article 6 of the Constitution of Kenya 2010. Nairobi, Kenya; 2010. http://kenyalaw.org/kl/index.php?id=398
2. MoH. Kenya Health Policy 2014 – 2030. Nairobi, Kenya; 2014. https://www.eahealth.org/policy-publications/kenya-health-policy-2014-%E2%80%93-2030
3. MoH. Nairobi; 2021 [updated 2021; cited 2021 10th May]; Available from: https://knh.or.ke/index.php/history/
4. Hensher M, Price M, Adomakoh S. Referral Hospitals. Disease Control Priorities in Developing Countries 2nd edition Washington (DC): The International Bank for Reconstruction and Development / The World Bank; 2006 2nd Edition ed. New York: Oxford University Press; 2006. p. 1229 - 43.
5. Holdsworth G, Garner PA, Harphan T. Crowded outpatient departments in city hospitals of developing countries: a case study from Lesotho. Int J Health Plann Manage. 1993 Oct-Dec;8(4):315-24.
6. London L, Bachmann OM. Paediatric utilisation of a teaching hospital and a community health centre. Predictors of level of care used by children from Khayelitsha, Cape Town. S Afr Med J. 1997 Jan;87(1):31-6.
7. Ohara K, Melendez V, Uehara N, Ohi G. Study of a patient referral system in the Republic of Honduras. Health Policy Plan. 1998 Dec;13(4):433-45.
8. Sanders D, Kravitz J, Lewin S, McKee M. Zimbabwe's hospital referral system: does it work? Health Policy Plan. 1998 Dec;13(4):359-70.

9. Stefanini A. District hospitals and strengthening referral systems in developing countries. World Hosp Health Serv. 1994;30(2):14-9.

10. KNH IN-PATIENT STATISTICS REPORT 2018, 2019 & 2020 [cited June 2020].

11. Casagrande JT, Pike MC, PG S. An improved approximation formula for calculating sample sizes for comparing two binomial distributions. Biometrics 1978; 34:483-6

12. Ahmed E, Chaka T. The pattern of orthopedic admissions in Tikur Anbessa University Hospital, Addis Ababa. Ethiop Med J. 2005 Apr;43(2):85-91.

13. Saikiran V, Laxmi PG, Nagaraju V, Bharath KD, Anchuri. SS. Epidemiology of orthopedic trauma admissions in a multispecialty hospital in Warangal-A retrospective study. Clinical Practice. 2019;16(6).

14. Mishra BN, Jha A, Gupta. Epidemiology of Orthopaedic Admissions at A Teaching Hospital of Eastern Nepal. Journal of Nobel Medical College. 2017;6(1):56–62.

15. Suraj B, Suresh P, Hemant KG, Roshani A, Bhattarai K. Pattern of Injury among Orthopaedic Inpatients in Teaching Hospital in Nepal. Journal of College of Medical Sciences-Nepal. Oct-Dec 2020;16(4).

16. Chandrashekara CM, George MA, Al-Marboi BSK. Demography of orthopaedic admissions in a secondary care hospital in oman. Brunei International Medical Journal 2013;9(4):236-42.

17. Premkumar A, Massawe HH, Mshabaha DJ, R.Foran J, XiaohanYing, Sheth NP. The burden of orthopaedic disease presenting to a referral hospital in northern Tanzania. Global Surgery. 2015;2(1):70-5.

18. Manwana ME, Mokone GG, Kebaetse M, T Y. Epidemiology of traumatic orthopaedic injuries at Princess Marina Hospital, Botswana. South African Orthopaedic Journal. 2018 March 2018;17(1):41-6.

19. Kihuba E. Epidemiology and medical costs of orthopedic conditions in a tertiary hospital in Kenya; A five-year analysis of admission data. BMJ Yale. 2022.

20. Pan RH, Chang NT, Chu D, Hsu KF, Hsu YN, Hsu JC, et al. Epidemiology of orthopedic fractures and other injuries among inpatients admitted due to traffic accidents: a 10-year nationwide survey in Taiwan. ScientificWorldJournal. 2014; 2014:637872.

21. Njanwe PJ, Marete I, Ayaya S. Adherence to national healthcare referral guidelines and its effect on the management outcomes among children seen at a Teaching Hospital in western Kenya. East African Medical Journal. 2021;98(1).

22. Lal S, Ndyomugenyi R, Paintain L, Alexander ND, Hansen KS, Magnussen P, et al. Community health workers adherence to referral guidelines: evidence from studies introducing RDTs in two malaria transmission settings in Uganda. Malar J. 2016 Nov 24;15(1):568.

23. Boissin C, Hasselberg M, Kronblad E, Kim SM, Wallis L, Rode H, et al. Adherence to Referral Criteria at Admission and Patient Management at a Specialized Burns Centre: The Case of the Red Cross War Memorial Children's Hospital in Cape Town, South Africa. Int J Environ Res Public Health. 2017 Jul 6;14(7).

24. Pittalis C, Brugha R, Bijlmakers L, Mwapasa G, Borgstein E, Gajewski J. Patterns, quality and appropriateness of surgical referrals in Malawi. Trop Med Int Health. 2020 Jul;25(7):824-33.

Captions of supporting Information files

S1 Figure 1: referral linkages between different levels of care (Reproduced from Kenya Health Sector Implementation Referral Guidelines, 2014)

S2 Table 1: Socio-demographic characteristics between orthopaedic and trauma admissions at KNH before and after enforcement of the referral guidelines, 2021

S3 Table 2: Walk-ins and Facility Referrals before and after enforcement of referral guidelines, 2021

S4 Table 3: Orthopaedic and trauma admissions to KNH stratified by point of admission, 2021.

S5 Table 4: Type of orthopaedic and trauma admissions before and after enforcement of referral guidelines, 2021

S6 Table 5: Facility referrals with referral letters before and after enforcement of referral guidelines, 2021

S7 Table 6: Multivariable analysis of socio-demographic characteristics of orthopaedic admissions to KNH before and after enforcement of referral guidelines, 2021

S8 Table 7: Multivariable analysis of sex of orthopaedic admissions to KNH before and after enforcement of referral guidelines, 2021 disaggregated by mode of payment

S9 Table 8: Association between age and point of admission before and after enforcement of referral guidelines stratified by Facility referral and Walk-ins, 2021

S10 Table 9: Association between the nature of injury before and after enforcement of referral guidelines, 2021.

S11 Supporting Information – Working dataset 20.5.2022

Other - ERB approval letter - ERC APPROVAL LETTER.pdf

 Click here to access/download;Figure;Fig 1.docx

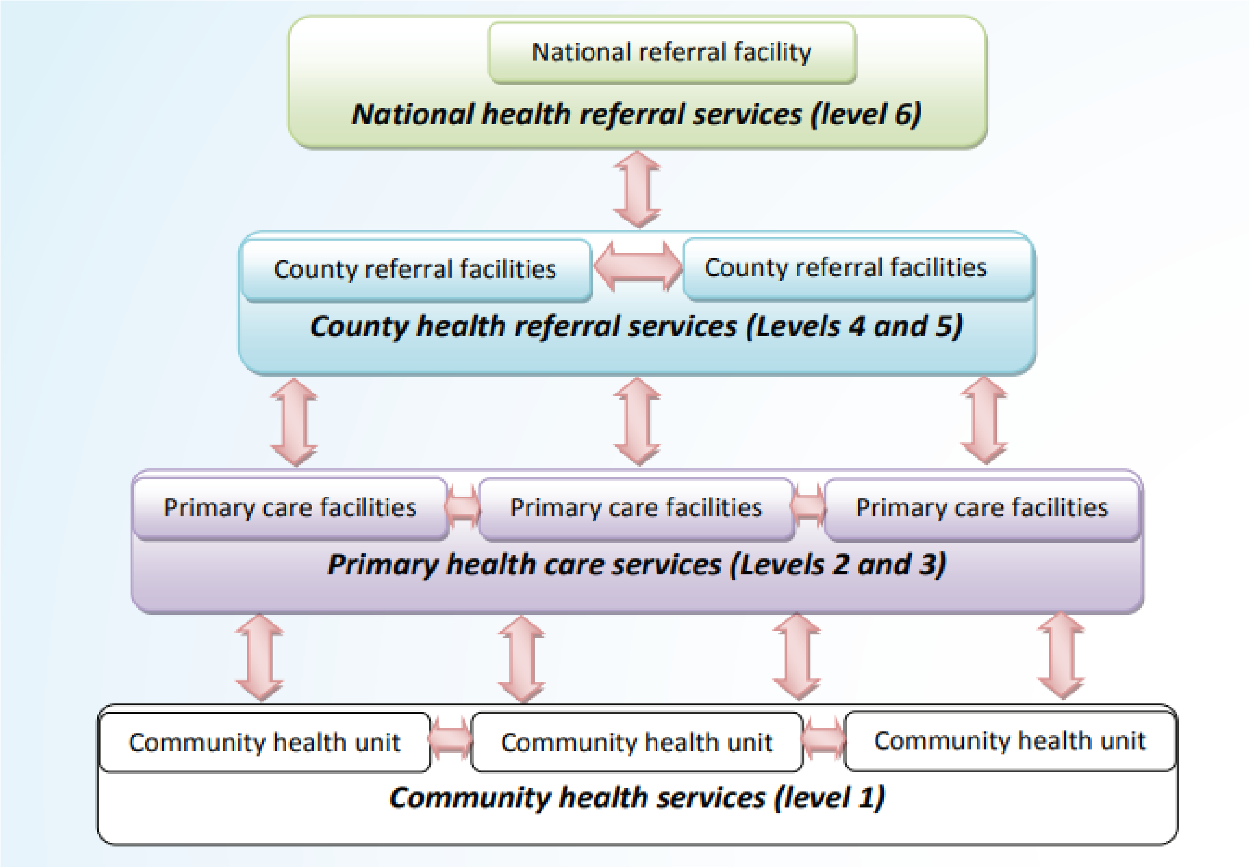

Figure 1: referral linkages between different levels of care (Reproduced from Kenya Health Sector Implementation Referral Guidelines, 2014)

Supporting Information S11

Click here to access/download
Supporting Information
WORKING. 05.06.2022.sav

Effect of enforcement of referral guidelines on patterns of orthopedic and trauma admissions to Kenyatta National Hospital, Kenya: pre-posttest study design

Authors: Maxwell Philip Omondi[1¶], Joseph Chege Mwangi[1¶], Fred Chuma Sitati[1¶], Herbert Onga'ngo'[2¶]

[1] Orthopedics Unit, Department of Surgery, University of Nairobi, Nairobi, Kenya
[2] Department of Orthopedics and Trauma, Kenyatta National Hospital, Nairobi, Kenya

*Corresponding author: Maxwell Philip Omondi
Email: maxwellomondi@gmail.com

¶All authors contributed equally to this work.

Abstract

Inappropriate utilization of higher-level health facilities and ineffective management of referral processes in resource-limited settings is increasingly becoming a concern in health care management in developing countries. This is characterized by self-referral and frequent bypassing of the nearest health facilities coupled with low formal referral mechanisms. This scenario lends itself to a situation where uncomplicated medical conditions are unnecessarily managed in a high-cost health facility. On July 1, 2021, Kenyatta National Hospital (KNH) did enforce the Kenya Health Sector Referral Implementation Guidelines, 2014 which required patients get approval from KNH referral office and a formal referral letter to be admitted at KNH to reduce the number of walk-ins

and allow KNH to function as a referral facility as envisioned by Kenya 2010 Constitution and KNH legal statue of 1987. The study objective was to determine the effect of enforcement of referral guidelines on patterns of orthopaedic and trauma admissions to KNH. This was a pre-post-test study design. Data abstraction was done for 459 and 446 charts before and after enforcement of referral guidelines. Enforcement of the referral guidelines reduced the proportion of walk-in admissions from 54.9% to 45.1%, while proportion of facility referrals increased from 46.6% to 53.4% ($p=0.013$). The Non-trauma orthopaedic admissions doubled from 12.0% to 22.4% ($p<0.001$). There was also a notable increase in admissions through the Outpatient Clinic (OC) and Corporate Outpatient Clinic (COC). The proportion of emergency admissions declined while that of elective admissions increased. The increase in elective cases were mainly driven by the increase in female admissions with active insurance cover, tertiary education, non-trauma related conditions and of older age groups. However, use of official formal written referral letters did not change despite the enforcement of referral guidelines. In conclusion, the enforcement of the referral guidelines reduced the proportion of walk-ins' admissions to KNH. The admissions had more non-trauma conditions, had tertiary education level, and more active insurance cover as a result of enforcement of the referral guidelines.

Keys: referral guidelines, Orthopaedic and trauma, pattern of admission

Background

Approximately 90% of the estimated traumatic injuries occur in low and middle-income countries according to World Health Organization (1) and this represents an important global public health problem now and in the coming years (2, 3).

Formatted: Highlight
Formatted: Highlight
Formatted: Highlight
Formatted: Highlight
Formatted: Highlight
Formatted: Highlight
Formatted: Highlight

Tertiary hospitals in resource-limited countries treat referred patients but in most cases are the first level of care for the vast majority of patients (4). One of the challenges in health care delivery in resource-limited settings is the inappropriate utilization of tertiary health facilities that results in patients' congestion in referral hospitals with simple conditions that can be effectively managed at lower health facilities. The majority of these patients are self-referred, bypassing lower-level health facilities in the process (5-8).

This jeopardizes the appropriate delivery of primary, secondary, and tertiary health care. Tertiary health care is compromised by the large demand for primary health care and in the process cripples the primary health care system and this effectively ensures that primary health care facilities remain underused and inefficient (9).

Kenya is administratively organized into forty-seven (47) counties with Nairobi Capital city being in Nairobi County (1). Health care service delivery has been devolved to the county level with the national level being responsible for health policy formulation and strategic planning. Kenyatta National Hospital (KNH) falls under national government while County and Subcounty level hospitals fall under County government administratively (1)(10) (2). The Kenya health service delivery system is organized across six levels of care,

Formatted: Highlight
Formatted: Highlight
Formatted: Highlight
Field Code Changed

beginning at the community level (level 1) and continuing through primary care services, which include dispensaries (level 2) and health centers (level 3), and county referral health services (level 4 & 5) all the way to the national referral health services (level 6) (See Fig 1) (2)(11). KNH is a level 6 facility and falls under national government as a premier national referral health facility which means it is a national tertiary level hospital in the Kenyan health system that offers very specialized skills, expertise and services (2)(11).

Figure 1: Rreferral linkages between different levels of care (Reproduced from Kenya Health Sector Implementation Referral Guidelines, 2014)

Kenyatta National Hospital (KNH) was established in 1901 as a National Referral and Teaching Hospital to provide training and medical research in Kenya. KNH became a State Corporation in 1987 and sits at the apex of the health referral system in Kenya (3)(12). According to the KNH Board order of 1987 contained in the Legal Notice No. 109, the functions of KNH were spelled out as a) to receive patients on referral for specialized health care; b) to provide facilities for medical education for the University of Nairobi and other health allied courses; c) to contribute to national health planning (3)(12). This understanding has been reinforced by the Kenya Health Sector Referral Implementation Guidelines, 2014, and the Constitution of Kenya 2010 which tasks KNH with the responsibility for health policy formulation (1, 2)(10, 11).

Field Code Changed

Field Code Changed

Field Code Changed

Tertiary hospitals in resource-limited countries treat referred patients but in most cases are the first level of care for the vast majority of patients (4). One of the challenges in health care delivery in resource-limited settings is the inappropriate utilization of tertiary health facilities that results in patients' congestion in referral hospitals with simple conditions that can be effectively managed at lower health facilities. The majority of these patients are self-referred, bypassing lower-level health facilities in the process (5-8). This jeopardizes the appropriate delivery of primary, secondary, and tertiary health care.

Tertiary health care is overwhelmed by the large demand for primary health care that should be managed at lower level health facilities and in the process cripples the primary health care system and this effectively ensures that primary health care facilities remain underused and inefficient (9).

Orthopaedic wards in KNH have consistently recorded the highest bed occupancy percent for the last couple of years. In 2018, 2019, and 2020, it recorded bed occupancy percent of 142.2%, 138.2%, and 116.5%, respectively, with the overall KNH bed occupancy percent of 106.2%, 113.4 percent and 91.5% (10)(13). This coupled with inadequate staffing levels results in e consequence of this is the low nurse-patient ratio of 1:10 that compromises not only the quality of nursing care given to patients but also compromises the ability of KNH to effectively perform its statutory obligations.

At the strategic level, the government's overall strategic plan, the Second Medium-Term Plan (MTP) of Vision 2030, identifies enforcement of referral guidelines as a key strategy

Formatted: Highlight

Formatted: Highlight

Formatted: Highlight

Field Code Changed

to increase the use of services at lower levels of the health care system and reduce self-referral to higher levels of care.

Consequently, on 1st July 2021, KNH management made a decision to reinforce the Kenya Health Sector Referral Guidelines 2014 that places KNH at the tip of the health sector referral system. This meant that patients will be seen based on referral letters from other health facilities to reduce the number of walk-in patients who would have otherwise been appropriately seen at the peripheral health facilities. This will contribute to the decongestion of KNH Orthopaedic and trauma admissions. The purpose of this study was to determine the effect of the enforcement of referral guidelines on the patterns of orthopaedic and trauma admissions to Kenyatta National Hospital.

Methods and Materials

Study design: This was a pre-post-test study design. Enforcement of referral guidelines required that the referring health facility consults with KNH referral coordination Office for concurrence before that patients are referred to KNH. This was is to ensure only a) patients who require specialized medical and orthopaedic and trauma care not available at the peripheral health facilities or b) no neighbouring government health facility has the capacity to manage the cases, get admitted to KNH.

Study area: KNH is the largest teaching and referral hospital in East and Central Africa. KNH Orthopaedic Wards were the study area. KNH is based in Upperhill, Nairobi, the capital city of Kenya. It is located along Hospital Road, about 5km from the city centre. KNH has a bed capacity of 1,800, 6,000+ staff members, 50 wards, 22 out-patient clinics, 24 theaters (16 specialized) and Accident & Emergency Department(3). Of the 1800 bed

Formatted: Font: (Default) Arial, 12 pt

Formatted: Font: (Default) Arial, 12 pt

capacity It has a bed capacity of approximately 2000 beds, 96 beds are allocated to orthopaedic .wards. KNH is a 10-floor storied building complex and the Orthopaedic wards are located on the 6th floor but we also have admissions in private wings in 9th and 10th floor. Orthopaedic patients with other co-morbidities also get admitted to other wards in KNH.

Study population: Orthopaedic in-patient case loads

Study duration: The study duration was from February 1st to December 31st 2021. It covered 5 months before and 5 months after enforcement of the referral guidelines, that is, from February 1, to June 30, 2021, before enforcement of the referral guidelines and from August 1, to December 31, 2021 after enforcement of the referral guidelines. The referral guidelines were enforced from July 1, 2021. Inclusion criteria was all orthopaedic and trauma admissions to KNH during the study period. 1st February to 31st December 2021. Data was accessed from the patient charts from 1st January to 31st March 2022 during the data collection period.

Sample size calculation and sampling procedure: Using an adjusted formula for calculating sample sizes that compare two binomial distributions (11)(14), a total of 459 files and 446 files were abstracted before and after the enforcement of the referral guidelines. Stratified sampling technique was used with orthopaedic and trauma admissions being stratified by month of admission and Population Proportionate to Size (PPS) was used to determine the sample size per strata. Systematic random sampling was then used to identify the sampled files using the KNH Health Records Register as the sampling frame from each of these three service entry points {Accident and Emergency (A&E), Orthopedic Clinic (OC), and Corporate Outpatient Clinic (COC)}. This

Formatted: Superscript

Formatted: Superscript

Formatted: Superscript

Field Code Changed

ensured the selected sample would be representative of the admissions by month from each of these three orthopedic and trauma admissions entry points. Informed consent, auditory, and visual confidentiality were provided.

Variables: variables abstracted for this study were a) Socio-demographic characteristics – age, sex, marital status, religion, education level b) Nature of admission; c) Occupation; d) Type of admission; e) Mode of payment;

Data collection procedures: Data collection was done through a data abstraction form. This was a manual survey tool. Data were abstracted from the patient charts and transferred to the data abstraction form by the research assistants.

Formatted: Justified

Quality assurance & quality control procedures: Three Research assistants underwent two (2) day training on the study protocol. A pilot study was conducted during the design of the study protocol to test the data collection tools for relevance, appropriateness to answer the research questions, and adjustments of the data collection tools made as necessary. The Principal Investigator conducted daily reviews of all abstracted forms, verified for accuracy, completeness, and compliance with the research protocol. Inaccurate, incomplete and non-compliance entries were repeated under the supervision of the Principal Investigator as part on ongoing training and to ensure similar errors are minimized.

Ethical considerations: UoN/KNH Ethics and Research Committee granted ethical approval (ERC No: P852/10/2021). Administrative approval was also sought from KNH Medical Research Department and KNH Orthopaedics Department. This study was carried out in accordance with UoN/KNH Ethics and Research Committee guidelines and

regulations. For the underage, formal verbal consent was obtained from the parent/guardian.

Data management and analysis plan: Data abstraction tool was used to collect quantitative data. The data were entered into a password-protected Redcap database. Descriptive analysis was done using measures of central tendency (mean), measures of dispersions such as range, standard deviation were used, frequency distribution were also used. Inferential analysis was done using Pearsons's chi-square test, logistic regression, odds ratio to determine effect of the enforcement of the referral guidelines and also determine possible associations. SPSS version 21 was used.

Study limitations: a) Incomplete and missing records – this was addressed by increasing the sample size by 10%; bc) Effect of COVID 19 pandemic on referrals of cases from peripheral health facilities and walk-in patients – this was addressed by ensuring the data collection period covered the covid period when the restrictions of intercounty movements were already lifted. Despite these limitations, given the paucity of published literature in this filed, this study offers key information on the effects of enforcement of referral guidelines in Orthopaedic and trauma admissions in KNH with important lessons for Kenya and possible sub-Saharan Africa.

Results

I) Socio-demographic characteristics of the sample population

Formatted: Numbered + Level: 1 + Numbering Style: I, II, III, … + Start at: 1 + Alignment: Left + Aligned at: 0.25" + Indent at: 0.75"

The total number of charts abstracted was 905 with 459 charts before and 446 charts after the enforcement of the referral guidelines.

Key socio-demographic characteristics of orthopaedic and trauma admissions at KNH such as age, sex, education, occupation, and marital status, smoking and alcohol

werestatus were reviewed and compared between the two groups. Majority of the admissions were aged between 25 – 64 years with children and elderly (above 65 years) represented the least admissions. Over three quarter of the admissions were male before and after the enforcement of referral guidelines. The male admissions peaked at 25 – 64 years of age and declined steadily to 65years of age and orthopaedic admissions were comparable across sex for those above 65 years of age. A good proportion of the admissions had primary and secondary level of education (Table 1).

Education and sex were noted to be statistically significant between the two groups at p=0.001 and p=0.005, respectively. However, age, religion, occupation, marital status, smoking, and alcohol intake habits were comparable amongst the two groups (P > 0.05) (Table 1).

Table 1: Socio-demographic characteristics between orthopaedic and trauma admissions at KNH before and after enforcement of the referral guidelines, 2021

Variable	Category	Before (n=459)	After (n=446)	Chi-square, p-value
Age	0 – 14 years	59 (59.6%)	40 (40.4%)	7.121 (p=0.068)
	15 – 24 years	83 (50.0%)	83 (50.0%)	
	25 – 64 years	303 (50.5%)	297 (49.5%)	
	Above 65 years	14 (35.0%)	26 (65.0%)	
Sex	Female	83 (41.9%)	115 (58.1%)	7.866 ***(p=0.005)***
	Male	374 (53.2%)	329 (46.8%)	
	Missing	2	2	
Marital status	Married	218 (48.9%)	228 (51.1%)	4.450 (p=0.349)
	Minor	61 (55.5%)	49 (44.5%)	
	Separated & divorced	20 (40.8%)	29 (59.2%)	
	Single	147 (53.5%)	128 (46.5%)	
	Widow	13 (54.2%)	11 (45.8%)	
	Missing		1	
Religion	Atheist	2 (66.7%)	1 (33.3%)	2.113 (p=0.609)
	Christian	433 (50.1%)	432 (49.9%)	
	Hindu	1 (33.3%)	2 (66.7%)	
	Muslim	15 (62.5%)	9 (37.5%)	
	Missing	8	2	
Occupation	Businessman/woman	60 (52.6%)	54 (47.4%)	1.416 (p=0.841)
	Casual	204 (50.6%)	199 (49.4%)	

	Employed	64 (47.4%)	71 (52.6%)	
	Other	33 (55.9%)	26 (44.1%)	
	unemployed	92 (50.3%)	91 (49.7%)	
	Missing	6	5	
Education	None	33 (60.0%)	22 (40.0%)	***0.001***
	Pre-school	18 (81.8%)	4 (18.2%)	
	Primary	157 (51.0%)	151 (49.0%)	
	Secondary	166 (52.4%)	151 (47.6%)	
	Tertiary	74 (40.7%)	108 (59.3%)	
	Missing	11	10	
Smoking	No	348 (51.2%)	332 (48.8%)	0.078 (p=0.780)
	Yes	89 (50.0%)	89 (50.0%)	
	Missing	22	25	
Alcohol	No	258 (50.1%)	257 (49.9%)	0.410 (p=0.522)
	Yes	180 (52.3%)	164 (47.7%)	
	Missing	21	25	

Legend: The table shows the independent categorical variables abstracted from the patient charts before and after enforcement of referral guidelines. Missing values ranged from 1 to 25 and were excluded from the analysis. Statistical analysis was determined by Pearson χ^2 statistics at 95% CI using SPSS version 21.0.

II) Effect of enforcement of the referral guidelines on orthopaedic and trauma admissions at KNH

Formatted: Numbered + Level: 1 + Numbering Style: I, II, III, … + Start at: 1 + Alignment: Left + Aligned at: 0.25" + Indent at: 0.75"

a) Profile of admissions to KNH

The effect of enforcement of the referral guidelines on profile of admissions namely nature of admissions (walk-ins vs facility referrals), point of admissions (A&E, OC, COC), types of admissions (emergency vs electives) and formal referrals were reviewed.

Formatted: Font: (Default) Arial, 12 pt

Formatted: Font: (Default) Arial, 12 pt

Formatted: Font: (Default) Arial, 12 pt

Formatted: Font: (Default) Arial, 12 pt

The proportion of walk-ins declined from 248/459 (54.9%) to 204/446 (45.1%) after the enforcement of the referral guidelines, while facility referrals increased from 211 (46.6%) to 242 (53.4%) during the same period (p=0.013) (Table 2). After controlling for possible confounders (sex and education level), the odds of walk-ins were 1.653 (95% CI: 1.244 – 2.197) as compared to facility referrals before the enforcement of referral guidelines This means the walk-ins were significantly high before the enforcement of the referral

guidelines and this reduced after the enforcement of the referral guidelines during the study period. This means that enforcement of the referral guidelines did reduce the proportion of admissions that were self-referred with a proportional increase in admissions that were facility referred.

Table 2: Walk-ins and Facility Referrals before and after enforcement of referral guidelines, 2021

	Before (n=459)	After (n=446)	Chi-square; p-value	Logistic regression
Walk-ins	248 (54.9%)	204 (45.1%)	***6.219; p=0.013***	***1.653 (95% CI: 1.244 – 2.197) (p=0.001)***
Facility referrals	211 (46.6%)	242 (53.4%)		

Legend: Pearson χ^2 statistics test was used to test for statistical significance at 95% CI and Odds Ratio Estimate was used to test the strength of association.

Although the majority of admissions were admitted through Accident and Emergency (A&E) department, there was a notable decline from 386 (84.1%) and 321 (72.0%) after enforcement of the referral guidelines (p=0.0001) with the least admissions through the Orthopaedic Clinics (OC). There was also a notable increase in admissions through Corporate Outpatient Care (COC) after enforcement of the referral guidelines from 48 (10.5%) to 87 (19.5%) (p=0.011) (Table 3). Overall, there was a significant decline in admissions through A&E and an increase in admissions thorough the COC and OC after the enforcement of the referral guidelines.

Table 3: Orthopaedic and trauma admissions to KNH stratified by point of admission, 2021.

	Before	After	Total	p-value
Accident & Emergency (A&E)	386 (84.1%)	321 (72.0%)	707 (78.1%)	***0.0001***
Corporate Outpatient Clinic (COC)	48 (10.5%)	87 (19.5%)	135 (14.9%)	***0.011***
Orthopaedic Clinics (OC)	25 (5.4%)	38 (8.5%)	63 (7.0%)	0.315
Total	459 (100.0%)	446 (100.0%)	905 (100.0%)	

Legend: Pearson χ^2 statistics test was used to test for statistical significance at 95% CI and Odds Ratio Estimate was used to test the strength of association.

The type of admissions was also reviewed before and after enforcement of referral guidelines. Emergency admissions declined from about 387 (84.7%) to 325 (73.4%), while elective cases increased from 70 (15.3%) to 118 (26.6%) %) (0.0001) after enforcement of the referral guidelines (Table 4). Elective admissions were 50.2% less likely to occur before as opposed to after enforcement of referral guidelines (Table 4). This means that elective admissions increased while emergency admissions reduced after the enforcement of the referral guidelines.

Table 4: Type of orthopaedic and trauma admissions before and after enforcement of referral guidelines, 2021

Characteristics		Before	After	Chi-square test; p-value	OR Estimate (95% CI)
Type of admission	Elective	70 (15.3%)	118 (26.6%)	***17.441; p=0.0001***	***0.498 (0.358 – 0.693)***
	Emergency	387 (84.7%)	325 (73.4%)		
	Total	457 (100.0%)	443 (100.0%)		

Legend: Pearson χ^2 statistics test was used to test for statistical significance at 95% CI and Odds Ratio was used to test the strength of association.

According to the Kenya Health Sector Referral Implementation Guidelines, 2014, facility referrals are expected to be accompanied by official written referral letters from the referring facilities to KNH. The study sought to determine the proportion of admissions accompanied by official written referral letters from the referring health facilities to KNHenforcement of this referral requirement. The study revealed the proportion of referrals with official written referral letters remained unchanged before and after enforcement of the referral guidelines (p=0.860) (Table 5). This means that the enforcement of the referral guidelines had no statistically significant effect on the proportion of admissions with written referral letters as required by the referral guidelines.

Table 5: Facility referrals with referral letters before and after enforcement of referral guidelines, 2021

		Facility referrals		Chi-square; p-value
		Before	After	
Have referral letters	No	109 (51.7%)	123 (50.8%)	0.031; p=0.860
	Yes	102 (48.3%)	119 (49.2%)	
Total		211 (100.0%)	242 (100.0%)	

Legend: Pearson χ^2 statistics test was used to test for statistical significance at 95% CI.

b) Key socio-demographic patterns of admissions to KNH

Binary Logistic regression was used to estimate the probability of occurrence of key socio-demographic patterns of admissions characteristics before and after the enforcement of the referral guidelines, namely, sex, education level, and mode of payment. The study revealed that the likelihood of being male was 1.575 (1.145 – 2.166) less likely more compared to females afterbefore the enforcement of the referral guidelines compared to those after the enforcement of the referral guidelines (0.005) (Table 6). This means that there were fewer male admissions after enforcement of the referral guidelines.

With regard to education level, the study demonstrated that the likelihood of having a tertiary level of education was about 54.4% more after less before the enforcement of the referral guidelines as opposed to after the enforcement of the referral guidelines (p=0.001) (Table 6). This means that more orthopedic and trauma admissions had a tertiary level of education after enforcement of the referral guidelines.

Regarding the mode of payment, the odds of being a cash payer were 1.846 (1.387 – 2.458) more before compared to after the enforcement of referral guidelines while the likelihood of having insurance cover was 46.2% less likely before enforcement of referral

Formatted: Line spacing: Double

Formatted: Font: (Default) Arial, 12 pt

Formatted: Font: (Default) Arial, 12 pt

guidelines compared to after enforcement of referral guidelines (Table 6). This means that the enforcement of the referral guidelines was associated with a statistically significant increase in the number of orthopedic and trauma admissions with active insurance cover.

Table 6: Multivariable analysis of key socio-demographic characteristics of orthopaedic admissions to KNH before and after enforcement of referral guidelines, 2021

Characteristics		Before	After	Chi-square test; p-value	Logistic regression (95% CI)
Sex	Female	83 (18.2%)	115 (25.9%)	7.866; p=0.005	1.0 (REF)
	Male	374 (81.8%)	329 (74.1%)		***1.575 (1.145 – 2.166)***
	Total	457 (50.7%)	444 (49.3%)		
Education level	None	33 (7.4%)	22 (5.0%)	18.128; p=0.001	1.0(REF)
	Pre-school	18 (4.0%)	4 (0.9%)		3.0 (0.894 – 10.063)
	Primary	157 (35.0%)	151(34.6%)		0.693 (0.387 - 1.243)
	Secondary	166 (37.1%)	151 (34.6%)		0.733 (0.409 – 1.313)
	Tertiary	74 (16.5%)	108 (24.8%)		***0.457 (0.247 – 0.845)***
	Total	448 (50.7%)	436 (49.3%)		
Mode of payment	Cash	344 (75.3%)	277 (62.2%)	17.837; p=0.0001	1.0(REF)
	Insurance	113 (24.7%)	168 (37.8%)		***0.538 (0.404 – 0.716)***
	Total	457 (50.7%)	445 (49.3%)		

Legend: Pearson χ^2 statistics test was used to test for statistical significance at 95% CI and Odds Ratio was used to test the strength of association. (REF) refers to reference group.

Formatted: Line spacing: Double

The mode of payment and its association with sex was reviewed and it showed no statistically significant association before the enforcement of the guidelines. However, males were 2.366 times more likely to be cash payers compared to females after

enforcement of the referral guidelines (Table 7). This means more females admissions had insurance cover after enforcement of referral guidelines.

Table 7: Multivariable analysis of sex of orthopaedic admissions to KNH before and after enforcement of referral guidelines, 2021 disaggregated by mode of payment

	Sex	Cash	Insurance	Chi-square, p-value	OR (95% CI)
Before	Female	58 (17.0%)	25 (22.1%)	1.519; p=0.218	1.0 (REF)
	Male	284 (83.0%)	88 (77.9%)		1.391 (0.822 – 2.355)
After	Female	54 (19.6%)	61 (36.5%)	***15.574; p=0.0001***	1.0 (REF)
	Male	222 (80.4%)	106 (63.5%)		***2.366 (1.534 – 3.649)***

Legend: Pearson χ^2 statistics test was used to test for statistical significance at 95% CI and Odds Ratio was used to test the strength of association. (REF) refers to reference group.

c) Association between patients' characteristics and nature of admissions

The effect of enforcement of the referral guidelines on patients characteristics (age, point of admissions and nature of injury) on the nature of admissions (walk-ins vs facility referrals) were reviewed.

The study revealed that amongst walk-ins, orthopaedicorthopedic and trauma admissions, those aged 15 – 24 years and 25 – 64 years had statistically significant decline in admissions through the A&E after enforcement of the guidelines (Table 8). This

Formatted: Font: (Default) Arial, 12 pt

Formatted: Font: (Default) Arial, 12 pt

means the enforcement of the referral guidelines were associated with less admissions in through A&E and more admissions through the OC and COC.

Table 8: Association between age and point of admission before and after enforcement of referral guidelines stratified by Facility referral and Walk-ins, 2021

	Age category	Point of admission	Before	After	Chi-square, p-value
Walk-ins	0 – 14 years	A&E	13 (72.2%)	5 (27.8%)	1.210; p=0.546
		Clinic	6 (54.5%)	5 (45.5%)	
		COC	5 (55.6%)	4 (44.4%)	
	15 – 24 years	A&E	49 (69.0%)	22 (31.0%)	***17.811; p=0.0001***
		Clinic	0 (0.0%)	6 (100.0%)	
		COC	2 (20.0%)	8 (80.0%)	
	25 – 64 years	A&E	129 (62.3%)	78 (37.7%)	***13.641; p=0.001***
		Clinic	11 (39.3%)	17 (60.7%)	
		COC	28 (40.0%)	42 (60.0%)	
	Above 65 years	A&E	3 (30.0%)	7 (70.0%)	0.932; p=0.628
		Clinic	0 (0.0%)	2 (100.0%)	
		COC	2 (20.0%)	8 (80.0%)	
Facility referral	0 – 14 years	A&E	30 (55.6%)	24 (44.4%)	0.687; p=0.709
		Clinic	3 (75.0%)	1 (25.0%)	
		COC	2 (66.7%)	1 (33.3%)	
	15 – 24 years	A&E	31 (40.8%)	45 (59.2%)	2.833; p=0.243
		Clinic	1 (100.0%)	0 (0.0%)	
		COC	0 (0.0%)	2 (100.0%)	
	25 – 64 years	A&E	123 (48.2%)	132 (51.8%)	5.169; p=0.075
		Clinic	4 (40.0%)	6 (60.0%)	
		COC	8 (26.7%)	22 (73.3%)	
	Above 65 years	A&E	8 (50.0%)	8 (50.0%)	2.00; p=0.368
		Clinic	0 (0.0%)	1 (100.0%)	
		COC	1 (100.0%)	0 (0.0%)	

Legend: Pearson χ^2 statistics test was used to test for statistical significance at 95% CI.

The study revealed a statistically significant increase in the proportion of non-trauma-related orthopaedic admissions after enforcement of the referral guidelines amongst walk-in admissions (p < 0.001). The odds of non-trauma-related admissions were 2.829 times higher afterbefore enforcement of referral guidelines compared to after enforcement of referral guidelines amongst walk-ins' admissions (Table 9). This means that there was a

statistically significant increase in non-trauma-related admissions after enforcement of the referral guidelines amongst walk-ins.

Table 9: Association between the nature of injury before and after enforcement of referral guidelines stratified by Facility referral and Walk-ins, 2021.

	Nature of Injury				
		Before	After	Chi-square, p-value	OR
Walk-in	Trauma related	204 (62.0%)	125 (38.0%)	***23.384; p <0.001***	***2.829 (1.842 – 4.345)***
	Non – Trauma related	45 (36.6%)	78 (63.4%)		
Facility Referral	Trauma related	200 (47.6%)	220 (52.4%)	2.095; p=0.148	1.736 (0.816 – 3.690)
	Non – Trauma related	11 (34.4%)	21 (65.6%)		

Legend: Pearson χ^2 statistics test was used to test for statistical significance at 95% CI and Odds Ratio was used to test the strength of association.

Formatted: Space Before: 0 pt

Discussions

I) Sociodemographic characteristics of the sample population

Formatted: Numbered + Level: 1 + Numbering Style: I, II, III, … + Start at: 1 + Alignment: Left + Aligned at: 0.25" + Indent at: 0.75"

The study demonstrated over three-quarter of orthopaedic and trauma admissions were male before and after enforcement of referral regulations during the study period. This compares favourably with studies done in Addis Ababa, India, Nepal, Middle East, and Botswana showed male predominates orthopaedic admissions (12-19)(15-22). In addition, male orthopaedic admissions predominated and peaked at 25 – 64 years, and these admissions declined steadily to 65years of age and orthopaedic admissions were comparable across sex for those above 65 years of age. This compares with a retrospective study done in a tertiary hospital in Nepal that showed similar admission rates from 60 years of age (14)(17).

Field Code Changed

Field Code Changed

Based on age groups, children and those above 65 years represented the least orthopaedic admissions during the study period before and after enforcement of the referral guidelines, while the majority of admissions were observed among the age group 25 – 64 years. This compares with a retrospective study on orthopaedic admissions done in Warangal, India, that showed the average age was 41.14 years. There was a bimodal distribution with high rates of admission for young adults up to the age of 35 years old as well as for those above 45 years old (13)(16). Similar studies also depicted low paediatric orthopaedic admissions (20)(23). However, this contrasts with a study done in PCEA Kikuyu Mission Hospital, Kenya, that showed 18.84% orthopaedic admissions were of paediatric age group (19)(22). This could be due to the fact that PCEA Kikuyu Mission Hospital is a private mission hospital and is an established and highly regarded specialized orthopaedic centre in the country.

Field Code Changed

Field Code Changed

However, the study revealed an overall increase in female orthopaedic admissions compared to males after the enforcement of referral guidelines. This can be attributed to reduction in the number of emergency admissions through Accident and Emergency department and the reduced number of admissions resulting from the road traffic accident due to the enforcement of the referral guidelines since the majority of these emergency admissions are male.

The enforcement of the referral guidelines was associated with more orthopedic and trauma admissions having a tertiary level of education, non-trauma related conditions and admissions with active insurance cover. These were may have been driven mainly by

admissions through the COC following the enforcement of the referral guidelines. COC is the private clinic wing of KNH.

II) Effect of the referral guidelines on orthopaedic and trauma admissions at KNH

Formatted: Numbered + Level: 1 + Numbering Style: I, II, III, … + Start at: 1 + Alignment: Left + Aligned at: 0.25" + Indent at: 0.75"

On 1st July 2021, KNH enforced referral guidelines that were meant to streamline the referral process from peripheral health facilities and allow KNH to a) manage complex orthopaedic and trauma cases that cannot be handled at lower-level health facilities as per the Kenya Health Sector Referral Implementation Guidelines, 2014 b) perform its statutory functions. This resulted in a statistically significant reduction of the proportion of walk-ins and an increase in proportion offacility referral cases. Patients were encouraged to seek services from lower-level health facilities and that they should come to KNH with a written formal referral letter from a lower-level health facility.

There was a significant decline in admissions through A&E and an increase in admissions thoroughthrough the COC and OC after the enforcement of the referral guidelines. This means the enforcement of the referral guidelines were associated with more admissions through COC and OC and less admissions through the A&E. And this is in tandem with notable reductions in the proportions of emergency admissions from 84.7% to 73.4% and a relative increase in the proportions of elective admissions. The enforcement of the referral guidelines was associated with more elective orthopaedic and trauma admissions and less emergency orthopaedic and trauma admissions, especially amongst walk-ins aged 15 – 64 years. Majority of these emergency admissions through A&E are young and middle-aged admissions which reported a significant decline after the enforcement

of the referral guidelines. The decline in emergency admissions reflect the fact that maybe these were now being handled at the lower-level facilities as opposed to being referred to KNH.

In addition, there was a statistically significant increase in non-trauma orthopaedic admissions doubling from 12.0% to 22.4% after enforcement of the referral guidelines, and this was a reinforcing the fact that elective orthopaedic admissions increased while emergency orthopaedic admissions reduced after the enforcement of the referral guidelines. Non- trauma orthopaedic cases are usually elective admissions.

The The enforcement enforcement of referral guidelines was associated with a statistically significant increase in the proportion of orthopaedic and trauma admissions with active insurance cover for those aged between 15 – 64 years amongst walk-ins' admissions. The increase in the proportion with insurance cover after the enforcement of the referral guidelines could be due to an increase in the number and proportion of female orthopedic admissions through the OC and COC. The increase in the elective cases were driven mostly by elderly women with degenerative musculoskeletal disorders who had tertiary level of education and had active insurance cover. These were admitted mostly through the COC. This is reinforcing the finding that the proportion of non-trauma related admissions increased after enforcement of the referral guidelines.

However, there was no significant difference when it came to facility referrals being accompanied by official written referral letters from the referring facilities to KNH. This

compares with study done in Moi Teaching and Referral Hospital on adherence to paediatric national referral protocol that revealed poor adherence with only 15.9% accounting for hospital referrals with a formal referral letters (21). A study in Uganda also revealed poor adherence to referral guidelines by Community Health Workers referring patients to health facilities (22). The low use of referral letters in the current study could be This is because most of the referrals were verbal over the telephone and once a verbal consensus had been reached, the referring health facilities did not see the need of writing a formal written referral letter. However, this contrasts with a study on adherence to referral guidelines and use of referral letters in Cape Town, South Africa and in Malawi that revealed over 90% adherence to referral guidelines (23, 24). This is likely due to the fact that Red Cross War Memorial Children's Hospital in Cape Town, South Africa and Queen Elizabeth Central Hospitals in Malawi were specialized treatment centres.

Conclusions

In conclusion, the enforcement of the referral guidelines significantly reduced the proportion of walk-in patients from 54.9% to 45.1%, while facility referrals increased from 46.6% to 53.4%. There was also a notable increase in orthopaedic and trauma admissions through the OC and COC after enforcement of the referral guidelines. The proportion of emergency admissions declined while that of elective admissions increased after the enforcement of the referral guidelines. The increase in elective cases were mainly driven by the increase in female admissions with active insurance cover, tertiary level of education, non-trauma related conditions and of older age groups. However, the use of official formal written referral letters did not change due to the enforcement of the referral guidelines.

Recommendations

Recommendations to KNH Orthopaedic Department

1. Strict compliance and adherence to the referral guidelines should continue to be enforced to allow KNH function as a premier referral facility and optimize the utilization of the lower-level health facilities;
2. Develop, disseminateion and train health workers on the referral guidelines and referral SOPs;
3. Design, disseminate standard referral tools, such referral forms and registers and enforce their routine usage;
4. Perform regular needs assessments for specialized services so as to be appraised of the actual capacity and limitations of the referring health facilities to facilitate smooth continuumous of care from the lower-level facilities to KNH;
5. Provide technical assistance and build capacity to strengthen the referral system at the county level to sustain the gains made.

Recommendations to County Health Management TeamGovernment

1. Ensure the availability of standard referral tools, such as referral forms, registers and other relevant forms for referrals at the facility level;
2. Ensure continuous supportive supervision and capacity building of county facilities in the referral system;
3. Ensure availability of accessible and high-quality health services in the county to reduce unnecessary referrals and allow timely and quality service provision.

Recommendations to Health facilities

1. Ensure the availability and utilization of standard referral tools, such as referral forms, registers and other relevant forms for referrals at the facility level.

Acknowledgements

We would like to sincerely acknowledge the work of Brian Okinyi and Micah J. Kipkemei for commitment and assistance in data collection process.

References

1. GoK. Article 6 of the Constitution of Kenya 2010. Nairobi, Kenya; 2010. http://kenyalaw.org/kl/index.php?id=398
2. MoH. Kenya Health Policy 2014 – 2030. Nairobi, Kenya; 2014. https://www.eahealth.org/policy-publications/kenya-health-policy-2014-%E2%80%93-2030
3. MoH. Nairobi; 2021 [updated 2021; cited 2021 10th May]; Available from: https://knh.or.ke/index.php/history/
4. Hensher M, Price M, Adomakoh S. Referral Hospitals. Disease Control Priorities in Developing Countries 2nd edition Washington (DC): The International Bank for Reconstruction and Development / The World Bank; 2006 2nd Edition ed. New York: Oxford University Press; 2006. p. 1229 - 43.
5. Holdsworth G, Garner PA, Harphan T. Crowded outpatient departments in city hospitals of developing countries: a case study from Lesotho. Int J Health Plann Manage. 1993 Oct-Dec;8(4):315-24.
6. London L, Bachmann OM. Paediatric utilisation of a teaching hospital and a community health centre. Predictors of level of care used by children from Khayelitsha, Cape Town. S Afr Med J. 1997 Jan;87(1):31-6.
7. Ohara K, Melendez V, Uehara N, Ohi G. Study of a patient referral system in the Republic of Honduras. Health Policy Plan. 1998 Dec;13(4):433-45.
8. Sanders D, Kravitz J, Lewin S, McKee M. Zimbabwe's hospital referral system: does it work? Health Policy Plan. 1998 Dec;13(4):359-70.
9. Stefanini A. District hospitals and strengthening referral systems in developing countries. World Hosp Health Serv. 1994;30(2):14-9.
10. KNH IN-PATIENT STATISTICS REPORT 2018, 2019 & 2020 [database on the Internet]. 2020 [cited June 2020].
11. Casagrande JT, Pike MC, PG S. An improved approximation formula for calculating sample sizes for comparing two binomial distribution. . Biometrics 1978;34:483-6

Formatted: Indent: Left: 0", First line: 0"

Formatted: Font: (Default) Calibri, 11 pt

Formatted: Font: (Default) Calibri, 11 pt

Formatted: Font: (Default) Calibri, 11 pt

12. Ahmed E, Chaka T. The pattern of orthopedic admissions in Tikur Anbessa University Hospital, Addis Ababa. Ethiop Med J. 2005 Apr;43(2):85-91.
13. Saikiran Velpula, Laxmi Prasanna Gummadi, Nagaraju Vallepu, Bharath Kumar Dasari, Anchuri. SS. Epidemiology of orthopedic trauma admissions in a multispecialty hospital in Warangal-A retrospective study. Clinical Practice. 2019;16(6).
14. Mishra BN, Jha A, Gupta. Epidemiology of Orthopaedic Admissions at A Teaching Hospital of Eastern Nepal. Journal of Nobel Medical College. 2017;6(1):56–62.
15. Suraj Bidary, Suresh Pandey, Hemant Kumar Gupta, Roshani Aryal, Bhattarai K. Pattern of Injury among Orthopaedic Inpatients in Teaching Hospital in Nepal. Journal of College of Medical Sciences-Nepal. Oct-Dec 2020;16(4).
16. Chandrashekara CM, George MA, Al-Marboi BSK. Demography of orthopaedic admissions in a secondary care hospital in oman. Brunei International Medical Journal 2013;9(4):236-42.
17. Premkumar A, Massawe HH, Mshabaha DJ, R.Foran J, XiaohanYing, Sheth NP. The burden of orthopaedic disease presenting to a referral hospital in northern Tanzania. Global Surgery. 2015;2(1):70-5.
18. Manwana ME, Mokone GG, Kebaetse M, T Y. Epidemiology of traumatic orthopaedic injuries at Princess Marina Hospital, Botswana. South African Orthopaedic Journal. 2018 March 2018;17(1):41-6.
19. Kihuba E. Epidemiology and medical costs of orthopedic conditions in a tertiary hospital in Kenya; A five-year analysis of admission data. BMJ Yale. 2022.
20. Pan RH, Chang NT, Chu D, Hsu KF, Hsu YN, Hsu JC, et al. Epidemiology of orthopedic fractures and other injuries among inpatients admitted due to traffic accidents: a 10-year nationwide survey in Taiwan. ScientificWorldJournal. 2014;2014:637872.
21. Njanwe PJ, Marete I, Ayaya S. Adherence to national healthcare referral guidelines and its effect on the management outcomes among children seen at a Teaching Hospital in western Kenya. East African Medical Journal. 2021;98(1).
22. Lal S, Ndyomugenyi R, Paintain L, Alexander ND, Hansen KS, Magnussen P, et al. Community health workers adherence to referral guidelines: evidence from studies introducing RDTs in two malaria transmission settings in Uganda. Malar J. 2016 Nov 24;15(1):568.
23. Boissin C, Hasselberg M, Kronblad E, Kim SM, Wallis L, Rode H, et al. Adherence to Referral Criteria at Admission and Patient Management at a Specialized Burns Centre: The Case of the Red Cross War Memorial Children's Hospital in Cape Town, South Africa. Int J Environ Res Public Health. 2017 Jul 6;14(7).
24. Pittalis C, Brugha R, Bijlmakers L, Mwapasa G, Borgstein E, Gajewski J. Patterns, quality and appropriateness of surgical referrals in Malawi. Trop Med Int Health. 2020 Jul;25(7):824-33.

1. Hanche-Olsen TP, Alemu L, Viste A, Wisborg T, Hansen KS. Trauma care in Africa: a status report from Botswana, guided by the World Health Organization's "Guidelines for Essential Trauma Care". World J Surg. 2012;36(10):2371-83.
2. Murray CJL, AD L. The Global Burden of Disease: A Comprehensive Assessment of Mortality and Disability From Diseases Injuries, and Risk Factors in 1990 and Projected to 20201996.
3. Murray CJL, AD L. Global Health Statistics: A Compendium of Incidence Prevalence and Mortality Estimates for Over 200 Conditions1996.
4. Hensher M, Price M, Adomakoh S. Referral Hospitals. 2006.
5. Holdsworth G, Garner PA, Harphan T. Crowded outpatient departments in city hospitals of developing countries: a case study from Lesotho. Int J Health Plann Manage. 1993;8(4):315-24.

Formatted: Font: (Default) Calibri, 11 pt, Highlight

Formatted: Font: (Default) Calibri, 11 pt

Formatted: Font: (Default) Calibri, 11 pt, Highlight

Formatted: Font: (Default) Calibri, 11 pt

Formatted: Indent: Left: 0", Hanging: 0.5", Line spacing: single

6. London L, Bachmann OM. Paediatric utilization of a teaching hospital and a community health center. Predictors of level of care used by children from Khayelitsha, Cape Town. S Afr Med J. 1997;87(1):31-6.
7. Ohara K, Melendez V, Uehara N, Ohi G. Study of a patient referral system in the Republic of Honduras. Health Policy Plan. 1998;13(4):433-45.
8. Sanders D, Kravitz J, Lewin S, McKee M. Zimbabwe's hospital referral system: does it work? Health Policy Plan. 1998;13(4):359-70.
9. Stefanini A. District hospitals and strengthening referral systems in developing countries. World Hosp Health Serv. 1994;30(2):14-9.
10. GoK. Article 6 of the Constitution of Kenya 2010. Nairobi, Kenya2010.
11. MoH. Kenya Health Policy 2014 – 2030. Nairobi, Kenya2014.
12. MoH. https://knh.or.ke/index.php/history/ Nairobi, Kenya2021 [
13. KNH IN-PATIENT STATISTICS REPORT 2018, 2019 & 2020 [Internet]. 2020 [cited June 2020].
14. Casagrande PaS. Biometrics 1978; 34:483-6
15. Ahmed E, Chaka T. The pattern of orthopedic admissions in Tikur Anbessa University Hospital, Addis Ababa. Ethiop Med J. 2005;43(2):85-91.
16. Saikiran Velpula, Laxmi Prasanna Gummadi, Nagaraju Vallepu, Bharath Kumar Dasari, Anchuri. SS. Epidemiology of orthopedic trauma admissions in a multispecialty hospital in Warangal-A retrospective study. Clinical Practice. 2019;16(6).
17. Mishra BN, Jha A, Gupta. Epidemiology of Orthopedic Admissions at A Teaching Hospital of Eastern Nepal. Journal of Nobel Medical College. 2017;6(1):56–62.
18. Suraj Bidary, Suresh Pandey, Hemant Kumar Gupta, Roshani Aryal, Bhattarai K. Pattern of Injury among Orthopedic Inpatients in Teaching Hospital in Nepal. Journal of College of Medical Sciences-Nepal. Oct-Dec 2020;16(4).
19. Chandrashekara CM, George MA, Al-Marboi BSK. Demography of orthopedic admissions in a secondary care hospital in oman. Brunei International Medical Journal 2013;9(4):236-42.
20. Premkumar A, Massawe HH, Mshabaha DJ, R.Foran J, XiaohanYing, Sheth NP. The burden of orthopedic disease presenting to a referral hospital in northern Tanzania. Global Surgery. 2015;2(1):70-5.
21. Manwana ME, Mokone GG, Kebaetse M, T Y. Epidemiology of traumatic orthopedic injuries at Princess Marina Hospital, Botswana. South African Orthopedic Journal. 2018;17(1):41-6.
22. Kihuba E. Epidemiology and medical costs of orthopedic conditions in a tertiary hospital in Kenya; A five-year analysis of admission data. BMJ Yale. 2022.
23. Pan RH, Chang NT, Chu D, Hsu KF, Hsu YN, Hsu JC, et al. Epidemiology of orthopedic fractures and other injuries among inpatients admitted due to traffic accidents: a 10-year nationwide survey in Taiwan. Scientific World Journal. 2014; 2014:637872.

Formatted: Indent: Left: 0", Hanging: 0.5"

Captions of supporting Information files

S1 Figure 1: referral linkages between different levels of care (Reproduced from Kenya Health Sector Implementation Referral Guidelines, 2014)

S2 Table 1: Socio-demographic characteristics between orthopaedic and trauma admissions at KNH before and after enforcement of the referral guidelines, 2021

S3 Table 2: Walk-ins and Facility Referrals before and after enforcement of referral guidelines, 2021

S4 Table 3: Orthopaedic and trauma admissions to KNH stratified by point of admission, 2021.

S5 Table 4: Type of orthopaedic and trauma admissions before and after enforcement of referral guidelines, 2021

S6 Table 5: Facility referrals with referral letters before and after enforcement of referral guidelines, 2021

S7 Table 6: Multivariable analysis of socio-demographic characteristics of orthopaedic admissions to KNH before and after enforcement of referral guidelines, 2021

S8 Table 7: Multivariable analysis of sex of orthopaedic admissions to KNH before and after enforcement of referral guidelines, 2021 disaggregated by mode of payment

S9 Table 8: Association between age and point of admission before and after enforcement of referral guidelines stratified by Facility referral and Walk-ins, 2021

S10 Table 9: Association between the nature of injury before and after enforcement of referral guidelines, 2021.

S11 Supporting Information – Working dataset 20.5.2022

Formatted: Not Highlight

Other - ERB approval letter - ERC APPROVAL LETTER.pdf

Formatted: Highlight

Comments	Response
Journal requirements	
1. Please ensure that your manuscript meets PLOS ONE's style requirements, including those for file naming	Noted
2. Thank you for stating in your Funding Statement:<br>"This study was partially funded by Kenyatta National Hospital (https://knh.or.ke/). MPO received the funding award. The research grant award was RFA 2020/21."<br>Please provide an amended statement that declares *all* the funding or sources of support (whether external or internal to your organization) received during this study, as detailed online in our guide for authors at http://journals.plos.org/plosone/s/submit-now. Please also include the statement "There was no additional external funding received for this study." in your updated Funding Statement.<br>Please include your amended Funding Statement within your cover letter. We will change the online submission form on your behalf.	Done. See the cover letter.
3. Please include captions for your Supporting Information files at the end of your manuscript, and update any in-text citations to match accordingly. Please see our Supporting Information guidelines for more information: http://journals.plos.org/plosone/s/supporting-information.	Done
4. We are unable to open your Supporting Information file WORKING. 05.06.2022.sav. Please kindly revise as necessary and re-upload.	Revised and uploaded. I have confirmed I am able to open the uploaded file in SPSS version 21.0
5. Please review your reference list to ensure that it is complete and correct. If you have cited papers that have been retracted, please include the rationale for doing so in the manuscript text, or remove these references and replace them with relevant current references. Any changes to the reference list should be mentioned in the rebuttal letter that accompanies your revised manuscript. If you need to cite a retracted article, indicate the article's retracted status in the References list and also include a citation and full reference for the retraction notice.	Done and added URL where necessary.<br>1. Retracted reference has been replaced accordingly. See reference #11 under the references section<br>2. Additional references added from reference #21 to #24 under the references section
Reviewer #1: Review	
Line 69-72 – The author states that tertiary health care is compromised by the large demand for primary health care and in the process cripples the primary healthcare system and ensure that the PHC facilities remain underutilized and inefficient.<br>This paragraph is contradictory and the author needs to restate the message for clarity.	Done. See Line 93 - 96
Line 75 – Nairobi City County is the correct name of 'Nairobi County'	The statement as worded is correct. I have not used the word Nairobi City County in my manuscript. "*Kenya is*

	administratively organized into forty-seven (47) counties with Nairobi Capital city being in Nairobi County" is the wording. See Line xxx
Line 74-83: could do with revision so that there is less repetition and clarity in the message	Done. See Line 60 - 61
Line 101-107: The author nearly concludes that the high bed occupancy is the single reason responsible for the low nurse-patient ratio. This may not be necessarily accurate, causality is not clearly demonstrated hence the need to reword this so that it is one of the possible reasons	Done. See Line 101 - 104
Line 114-121 – Is there any data on self referral for orthopedic cases, what is the acceptable self-referral rate? And what are the dangers to the health system of this? I would have liked to hear just a little bit more on this in the Introduction section.	There is paucity of literature on this topic for orthopedic cases. Patients seen at KNH should only be referred with a referral letter and that's why it is called a referral health facility. We should not have self-referred/walk-in patients. That's should be practice as per Kenya Health Sector Referral Implementation Guidelines 2014.<br>The dangers have already been captured in the introduction section – e.g., low nurse-patient ration due to overcrowding and this compromises the quality of care and prevents KNH to actualize its statutory mandate as outlined in the background. When there is overcrowding the KNH ends up spending more time, resources doing what should have been done at peripheral health facilities and therefore unable to effectively perform its statutory functions.
Overall comments:<br>The introduction section needs to be reworked a bit to tell the story of the author. As it is, it states facts but they do not flow very well into each other to tell the story.	Revised and edited to flow
Methods and materials	
Line 125 – '…. concurrence before that patients are referred to KNH.' This has a grammatical error	Edited. See Line 119
Line 125-127: 'This is to ensure only a) patients who require specialized medical and orthopedic and trauma 126 care or b) no neighboring government health facility has the capacity to manage the cases, get admitted to KNH.' This requires rewriting to avoid grammatical errors and make it clear	Edited. See Line 119 - 121
Study area	
Line 128-131: Needs more detail, how many floors does the hospital sit on? Staff establishment? Of the 2000 beds, how many are allocated to the orthopedic ward? This would be a	Done. See Line 122 - 133

good section to introduce the orthopedic wards in detail: talk about the bed capacity and the staffing issues and any other contextual information that would make the reader understand the state	
Line 133-140: Need to be reworded to be more concise. There is a bit of repetition especially on the date of guideline enforcement and. Once you state the date and the review duration, you will not need to granulate it further like you have.	Done. See Line 122 - 130
Line 156-157: Please describe the data collection tool more, was it an electronic or manual one? How were data handled if it was manual?	Done. See Line 153 -155
Line 158-164: What happened in the event of inaccuracy, incompleteness or non-compliance?	Done. See Line 162 - 164
Line 178-181: section (b) is missing. The effect of COVID is not clearly stated and the mitigation measures not understood. COVID probably decreased the number of referrals due to many reasons (restrictions in movement, change of SOP in hospitals etc.). Additionally, it would be good to contextualize the current state of the pandemic at the time of data collection since there were several phases characterized by variation sin regulations at every phase. i.e., at some point, restrictions were lifted, referrals were allowed etc. So at what point were we during the data collection.	Edited. See Line 178 - 186
Results Section	
The first statement should include the total number of cases included in the study. This is missing.	Done. See Line 189 - 190
Line 183-189: The text goes straight into multivariable analysis and we do not get to hear a little more description of the various socio-demographic variables in the text.	Done. See Line 191 - 199
Line 198: Proportion of walk ins – what is the denominator? This needs to be stated here	Done. See Line 229
Line 203-205: This requires to be re-interpreted and stated. As is, it is not very clear	Done. See Line 236 - 238
Line 238-245: Part of this paragraph sounds like a justification for the study or a discussion. May be best to simply report the results for this section and move the other descriptions to the their more appropriate sections	Done. See Line 272 -273
Line 249: The Legend seems to have become part of the paragraph (and I suspect that this was not the intention)	Formatted. See Table 5
Line 258-262: The interpretation appears to be incorrect	Edited. See Line 291
Line 306: Is that really true? That because 'The odds of trauma-related admissions were 2.829 times higher before enforcement of referral guidelines compared to after enforcement of referral guidelines amongst walk-ins' admissions' then 'This means that there was a statistically significant increase in non-trauma-related admissions after enforcement of the referral guidelines amongst walk-ins. '. I doubt that this statements are the mirror opposite of each other. There are probably confounders affecting this result.	Revised. See Line 338 -339

Line 319-324: these are the sentences I expected to see in the results' section i.i an account of the findings and then in the discussion we would have discussed these findings, compared and contrasted them with similar studies. Otherwise, this is like a new introduction of data that has not been previously reported elsewhere	Noted.
Line 336: the probable major reason is that PCEA Kikuyu is a specialized centre and not really that it is a private mission hospital	Edited. See Line 336
Line 338-343: That disparity in the number/proportion of female admissions cannot be quite explained by the enforcement of the guidelines. Let's dig deeper for some feasible reasons for this.	Noted. The enforcement reduced the number and proportion of emergency admissions. Emergency admissions were predominantly male.
Line 345-348: An association is bipartite between a dependent and independent variable. Your discussion tends towards a tripartite description i.e. enforcement of guidelines, orthopedic trauma and a 3rd factor etc. This needs to be refined so it is clear which factors are being evaluated against which variable	Noted
Line 355-357: needs to be reworded to be a bit 'tighter', esp line 356.	Edited. See Line 389 - 390
Line 360: thorough should be through	Edited. See Line 395
Line 361-363: we need to be clear on the dependent vs independent variable	Noted. The primary dependent variable is nature of admissions (walk ins vs facility referrals). The others are secondary variables
Line 372-376: these appear misplaced and do not quite communicate	Noted. Clarified. See Line 411
Line 378-387: This paragraph has 2 themes: comparing the association in the increase in trauma admissions with insurance with enforcement of guidelines. At Line 384, the other opines that this increase could be due to increase in the number and proportion of female cases. The conclusion that this is linked with the fact that these women had an active insurance cover may need a bit more of argument.	Noted. This is inferential since there was an increase in female admission after enforcement of the referral guidelines and also there was increase in those with active insurance cover after enforcement of referral guidelines. This is what the analysis revealed from the dataset shared. S11 Supporting Information – Working dataset 20.5.2022
Line 384, the other opines that this increase could be due to increase in the number and proportion of female cases. The conclusion that this is linked with the fact that these women had an active insurance cover may need a bit more of argument.	The analysis shows women were more likely to have insurance cover than men. It also showed an increase in women admissions above 65 years of age post enforcement of referral guidelines.
Line 389-393: Is there a result that communicates this consensus?	This is my expert opinion based on my understanding of the situation. I also have a related study that is under review in a PLOS Global Public Health journal that reinforces this understanding as outlined in the cover letter.

Recommendations	
They need to have a specific individual or office actioned otherwise they are not quite measurable	Noted. See recommendations section
Reviewer #2: The study topic is relevant and tries to address current concerns about referral procedures and pathways. The results are logical but the author should;	Noted
1. Remove un-important sections in the background. I found one particular paragraph not linking up	Revised and edited. See background section
2. Adjust and give more information about the study area and the study population	Done. See the revised manuscript.
3. Make result areas more clear. You can create small sub headings for easy flow	Done. See the results section of the revised manuscript
4. Only discuss significant findings.	Noted
5. Where possible, the author should try and give their interpretation of the trends.	Noted
5. There is need to find more literature to relate to or support the findings. Most of the findings in this study are not supported by other literature!	Noted. I have added 4 new references to support the discussions. However, there is paucity of published literature on this subject area specifically looking at the effect of referrals on hospitals admissions. This is a study of its kind.
