# Supplementary material for: Effect of enforcement of the national referral guidelines on patterns of orthopedic admissions to Kenyatta National Hospital, Kenya: Pre-post intervention study
Source: PLoS One. 2024 Aug 13;19(8):e0290195. doi: 10.1371/journal.pone.0290195 (PMC11321550; doi:10.1371/journal.pone.0290195)
Supplement: Attachment — Submitted filename: Response to Reviewers.docx [file pone.0290195.s004.docx]

| Comments | Response |
| --- | --- |
| Journal requirements |  |
| 1. Please ensure that your manuscript meets PLOS ONE's style requirements, including those for file naming | Noted |
| 2. Thank you for stating in your Funding Statement:  "This study was partially funded by Kenyatta National Hospital (<https://knh.or.ke/>). MPO received the funding award. The research grant award was RFA 2020/21."  Please provide an amended statement that declares *all* the funding or sources of support (whether external or internal to your organization) received during this study, as detailed online in our guide for authors at <http://journals.plos.org/plosone/s/submit-now>.  Please also include the statement “There was no additional external funding received for this study.” in your updated Funding Statement.  Please include your amended Funding Statement within your cover letter. We will change the online submission form on your behalf. | Done. See the cover letter. |
| 3. Please include captions for your Supporting Information files at the end of your manuscript, and update any in-text citations to match accordingly. Please see our Supporting Information guidelines for more information: <http://journals.plos.org/plosone/s/supporting-information>. | Done |
| 4. We are unable to open your Supporting Information file WORKING. 05.06.2022.sav. Please kindly revise as necessary and re-upload. | Revised and uploaded. I have confirmed I am able to open the uploaded file in SPSS version 21.0 |
| 5. Please review your reference list to ensure that it is complete and correct. If you have cited papers that have been retracted, please include the rationale for doing so in the manuscript text, or remove these references and replace them with relevant current references. Any changes to the reference list should be mentioned in the rebuttal letter that accompanies your revised manuscript. If you need to cite a retracted article, indicate the article’s retracted status in the References list and also include a citation and full reference for the retraction notice. | Done and added URL where necessary.   1. Retracted reference has been replaced accordingly. See reference #11 under the references section 2. Additional references added from reference #21 to #24 under the references section |
| Reviewer #1: Review |  |
| Line 69-72 – The author states that tertiary health care is compromised by the large demand for primary health care and in the process cripples the primary healthcare system and ensure that the PHC facilities remain underutilized and inefficient. This paragraph is contradictory and the author needs to restate the message for clarity. | Done. See Line 93 - 96 |
| Line 75 – Nairobi City County is the correct name of ‘Nairobi County’ | The statement as worded is correct. I have not used the word Nairobi City County in my manuscript. “*Kenya is administratively organized into forty-seven (47) counties with Nairobi Capital city being in Nairobi County*” is the wording. See Line xxx |
| Line 74-83: could do with revision so that there is less repetition and clarity in the message | Done. See Line 60 - 61 |
| Line 101-107: The author nearly concludes that the high bed occupancy is the single reason responsible for the low nurse-patient ratio. This may not be necessarily accurate, causality is not clearly demonstrated hence the need to reword this so that it is one of the possible reasons | Done. See Line 101 - 104 |
| Line 114-121 – Is there any data on self referral for orthopedic cases, what is the acceptable self-referral rate? And what are the dangers to the health system of this? I would have liked to hear just a little bit more on this in the Introduction section. | There is paucity of literature on this topic for orthopedic cases. Patients seen at KNH should only be referred with a referral letter and that’s why it is called a referral health facility. We should not have self-referred/walk-in patients. That’s should be practice as per Kenya Health Sector Referral Implementation Guidelines 2014.  The dangers have already been captured in the introduction section – e.g., low nurse-patient ration due to overcrowding and this compromises the quality of care and prevents KNH to actualize its statutory mandate as outlined in the background. When there is overcrowding the KNH ends up spending more time, resources doing what should have been done at peripheral health facilities and therefore unable to effectively perform its statutory functions. |
| Overall comments: The introduction section needs to be reworked a bit to tell the story of the author. As it is, it states facts but they do not flow very well into each other to tell the story. | Revised and edited to flow |
| Methods and materials |  |
| Line 125 – ‘…. concurrence before that patients are referred to KNH.’ This has a grammatical error | Edited. See Line 119 |
| Line 125-127: ‘This is to ensure only a) patients who require specialized medical and orthopedic and trauma 126 care or b) no neighboring government health facility has the capacity to manage the cases, get admitted to KNH.’ This requires rewriting to avoid grammatical errors and make it clear | Edited. See Line 119 - 121 |
| Study area |  |
| Line 128-131: Needs more detail, how many floors does the hospital sit on? Staff establishment? Of the 2000 beds, how many are allocated to the orthopedic ward? This would be a good section to introduce the orthopedic wards in detail: talk about the bed capacity and the staffing issues and any other contextual information that would make the reader understand the state | Done. See Line 122 - 133 |
| Line 133-140: Need to be reworded to be more concise. There is a bit of repetition especially on the date of guideline enforcement and. Once you state the date and the review duration, you will not need to granulate it further like you have. | Done. See Line 122 - 130 |
| Line 156-157: Please describe the data collection tool more, was it an electronic or manual one? How were data handled if it was manual? | Done. See Line 153 -155 |
| Line 158-164: What happened in the event of inaccuracy, incompleteness or non-compliance? | Done. See Line 162 - 164 |
| Line 178-181: section (b) is missing. The effect of COVID is not clearly stated and the mitigation measures not understood. COVID probably decreased the number of referrals due to many reasons (restrictions in movement, change of SOP in hospitals etc.). Additionally, it would be good to contextualize the current state of the pandemic at the time of data collection since there were several phases characterized by variation sin regulations at every phase. i.e., at some point, restrictions were lifted, referrals were allowed etc. So at what point were we during the data collection. | Edited. See Line 178 - 186 |
| Results Section |  |
| The first statement should include the total number of cases included in the study. This is missing. | Done. See Line 189 - 190 |
| Line 183-189: The text goes straight into multivariable analysis and we do not get to hear a little more description of the various socio-demographic variables in the text. | Done. See Line 191 - 199 |
| Line 198: Proportion of walk ins – what is the denominator? This needs to be stated here | Done. See Line 229 |
| Line 203-205: This requires to be re-interpreted and stated. As is, it is not very clear | Done. See Line 236 - 238 |
| Line 238-245: Part of this paragraph sounds like a justification for the study or a discussion. May be best to simply report the results for this section and move the other descriptions to the their more appropriate sections | Done. See Line 272 -273 |
| Line 249: The Legend seems to have become part of the paragraph (and I suspect that this was not the intention) | Formatted. See Table 5 |
| Line 258-262: The interpretation appears to be incorrect | Edited. See Line 291 |
| Line 306: Is that really true? That because ‘The odds of trauma-related admissions were 2.829 times higher before enforcement of referral guidelines compared to after enforcement of referral guidelines amongst walk-ins’ admissions’ then ‘This means that there was a statistically significant increase in non-trauma-related admissions after enforcement of the referral guidelines amongst walk-ins. ’. I doubt that this statements are the mirror opposite of each other. There are probably confounders affecting this result. | Revised. See Line 338 -339 |
| Line 319-324: these are the sentences I expected to see in the results’ section i.i an account of the findings and then in the discussion we would have discussed these findings, compared and contrasted them with similar studies. Otherwise, this is like a new introduction of data that has not been previously reported elsewhere | Noted. |
| Line 336: the probable major reason is that PCEA Kikuyu is a specialized centre and not really that it is a private mission hospital | Edited. See Line 336 |
| Line 338-343: That disparity in the number/proportion of female admissions cannot be quite explained by the enforcement of the guidelines. Let’s dig deeper for some feasible reasons for this. | Noted. The enforcement reduced the number and proportion of emergency admissions. Emergency admissions were predominantly male. |
| Line 345-348: An association is bipartite between a dependent and independent variable. Your discussion tends towards a tripartite description i.e. enforcement of guidelines, orthopedic trauma and a 3rd factor etc. This needs to be refined so it is clear which factors are being evaluated against which variable | Noted |
| Line 355-357: needs to be reworded to be a bit ‘tighter’, esp line 356. | Edited. See Line 389 - 390 |
| Line 360: thorough should be through | Edited. See Line 395 |
| Line 361-363: we need to be clear on the dependent vs independent variable | Noted. The primary dependent variable is nature of admissions (walk ins vs facility referrals). The others are secondary variables |
| Line 372-376: these appear misplaced and do not quite communicate | Noted. Clarified. See Line 411 |
| Line 378-387: This paragraph has 2 themes: comparing the association in the increase in trauma admissions with insurance with enforcement of guidelines. At Line 384, the other opines that this increase could be due to increase in the number and proportion of female cases. The conclusion that this is linked with the fact that these women had an active insurance cover may need a bit more of argument. | Noted. This is inferential since there was an increase in female admission after enforcement of the referral guidelines and also there was increase in those with active insurance cover after enforcement of referral guidelines. This is what the analysis revealed from the dataset shared. S11 Supporting Information – Working dataset 20.5.2022 |
| Line 384, the other opines that this increase could be due to increase in the number and proportion of female cases. The conclusion that this is linked with the fact that these women had an active insurance cover may need a bit more of argument. | The analysis shows women were more likely to have insurance cover than men. It also showed an increase in women admissions above 65 years of age post enforcement of referral guidelines. |
| Line 389-393: Is there a result that communicates this consensus? | This is my expert opinion based on my understanding of the situation. I also have a related study that is under review in a PLOS Global Public Health journal that reinforces this understanding as outlined in the cover letter. |
| Recommendations |  |
| They need to have a specific individual or office actioned otherwise they are not quite measurable | Noted. See recommendations section |
|  |  |
| Reviewer #2: The study topic is relevant and tries to address current concerns about referral procedures and pathways. The results are logical but the author should; | Noted |
| 1. Remove un-important sections in the background. I found one particular paragraph not linking up | Revised and edited. See background section |
| 2. Adjust and give more information about the study area and the study population | Done. See the revised manuscript. |
| 3. Make result areas more clear. You can create small sub headings for easy flow | Done. See the results section of the revised manuscript |
| 4. Only discuss significant findings. | Noted |
| 5. Where possible, the author should try and give their interpretation of the trends. | Noted |
| 5. There is need to find more literature to relate to or support the findings. Most of the findings in this study are not supported by other literature! | Noted. I have added 4 new references to support the discussions. However, there is paucity of published literature on this subject area specifically looking at the effect of referrals on hospitals admissions. This is a study of its kind. |
