# Supplementary material for: Effect of enforcement of the national referral guidelines on patterns of orthopedic admissions to Kenyatta National Hospital, Kenya: Pre-post intervention study
Source: PLoS One. 2024 Aug 13;19(8):e0290195. doi: 10.1371/journal.pone.0290195 (PMC11321550; doi:10.1371/journal.pone.0290195)
Supplement: Attachment — Submitted filename: Response to Reviewers 2.docx [file pone.0290195.s006.docx]

| Comments | Response |
| --- | --- |
| **ACADEMIC EDITOR:** |  |
| **Study design**: "This was a pre-post study design." This statement describing the study design is inadequate. Could the authors provide more details about the study design, such as what period was compared with what period and what was compared. | Revised. See marked up copy |
| **Study setting** (referred to as "study area" in the manuscript): While the authors describe the geographic location and physical infrastructure at KNH, crucial information about the current referral protocols in Kenya and KNH, vital for understanding the study context, is absent. To clarify the setting for the reader, please provide details on how patients navigate Kenya's healthcare system to reach KNH and the specific referral practices and guidelines in place at the hospital itself. Please also include information on how the referral guidelines were enforced and when this enforcement began. | Revised. See marked up copy |
| **Limitations:** These have been included in the methods section. Please discuss your study limitations in the discussion section. | Revised. See marked up copy |
| **Results: This section includes information that belongs to the methods and discussion sections, such as "**Key socio-demographic characteristics of orthopedic and trauma admissions at KNH such as age, sex, education, occupation, marital status, smoking and alcohol status were reviewed and compared between the two groups."  "This means that there was a statistically significant increase in non-trauma-related admissions after enforcement of the referral guidelines amongst walk-ins."  Revise this section thoroughly to remove all references to study methods or discussion. In this section only present your results. No methods, no discussion.  Tables - please revise the formatting of all tables to make them visually appealing and less crowded. Several tables presenting stratified analyses (Table 2 to 5) each contain only one variable. It is not clear why the authors decided to present it this way. A preferable way would be to include all these variables in one table. | Revised. See the results section in marked up copy  Revised. Merged Tables 2 – 4.  Table 5 has a different profile of messaging and therefore did not merge it to the others  Formatted all the tables |
| Reviewer #1: |  |
| The author has effected the revisions as requested with a clear description of the bed capacity and orthopedic case load. The results section begins with a summative response of the total cases studied. The scientific language is also tightened. The recommendations are now specific, and actioned to various entities | Noted with thanks |
| Reviewer #2: I reject the manuscript. |  |
| 1. The manuscript is not up to standard for publication in this journal as it is. The revisions made are not adequate. | Thanks for the comments. |
| 3. The author has not adequately supported the findings in this study. No much is done to relate the findings with previous related studies | Added more literature on previous studies. See marked up copy |
| 4. There is need to refine the flow and grammer in the entire manuscript- the author to avoid romans and bullets, exhaustively discuss and the findings, make precise and specific recommendations. | Edited the flow, removed the romans and bullets. Corrected typographical errors and grammer in the entire manuscript. Discussed the findings with more literature from previous studies. Reviewed the recommendations to make them more specific |
