# Supplementary material for: Effect of enforcement of the national referral guidelines on patterns of orthopedic admissions to Kenyatta National Hospital, Kenya: Pre-post intervention study
Source: PLoS One. 2024 Aug 13;19(8):e0290195. doi: 10.1371/journal.pone.0290195 (PMC11321550; doi:10.1371/journal.pone.0290195)
Supplement: Attachment — Submitted filename: Response to Reviewers 3.docx [file pone.0290195.s007.docx]

| Comments | Response |
| --- | --- |
| Journal Requirements: |  |
| Please review your reference list to ensure that it is complete and correct. If you have cited papers that have been retracted, please include the rationale for doing so in the manuscript text, or remove these references and replace them with relevant current references. Any changes to the reference list should be mentioned in the rebuttal letter that accompanies your revised manuscript. If you need to cite a retracted article, indicate the article’s retracted status in the References list and also include a citation and full reference for the retraction notice. | References reviewed and are complete and correct. |
| Reviewer #1: |  |
| The author has done a commendable job to revise the manuscript and it now reads better. The various sections do not have misplaced information, the manuscript is nor summarized better and the recommendations provided are specific. However we have an opportunity to improve. I provide the following comments and recommendations:  Line 112-113 – requires grammatical edits | Noted  Edits made. See the Manuscript with track changes |
| Line 118-121 – move the detailed timelines to study duration section below (line 160) so that there is no repetition | Edited. See the Manuscript with Track changes |
| Line 150 – needs to be edited to read ‘all patients being referred to KNH…’ | Edited. |
| Line 151-156 – is quite repetitive, could be summarized more concisely to communicate that 1) a decision was made, 2) it was enforced and 3) it was communicated (to whom and how) | Edited |
| Line 180 – from whom was informed consent sought since this was a retrospective study? How did we seek auditory and visual consent? This may need to be moved to the paragraph on ethical considerations | Deleted since it was a retrospective study and authors did chart reviews. |
| Line 273-274 –May be better to reword this or altogether move this line to the interpretation/discussion section. | Edited |
| Line 279 – delete the word ‘notable’ and state the result as is | Edited |
| Line 281 - delete the word ‘notable’ and state the result as is | Edited |
| Line 345 - delete the word ‘about’ | Edited |
| Reviewer #2: |  |
| The author has tried to address the issues raised and has related his finding to other previous researches despite the study area having limited literature. | Noted |
|  |  |
|  |  |
