# Supplementary material for: Effect of enforcement of the national referral guidelines on patterns of orthopedic admissions to Kenyatta National Hospital, Kenya: Pre-post intervention study
Source: PLoS One. 2024 Aug 13;19(8):e0290195. doi: 10.1371/journal.pone.0290195 (PMC11321550; doi:10.1371/journal.pone.0290195)
Supplement: Attachment — Submitted filename: Response to Reviewers 4.docx [file pone.0290195.s008.docx]

| Comments | Response |
| --- | --- |
| **ACADEMIC EDITOR:** |  |
| **1 - The manuscript has numerous grammatical errors. Thorough editing for langauage is needed. Below is an example:**  **- "Kenyatta National Hospital (KNH) are managed by the national government ..."**  **- There are some long and winding statements that are not easy to follow. The authors could revise to use clear and concise statements.**  **- line 96 to 99 is a very short paragraph that should be combined with the preceding paragraph** | Edited. See page 3, Line 69  Edited across the manuscript in track changes.  Revised. See page 4, See lines 93 -100 |
| **2 - Repetition - The concept of KNH being a national referral hospital is repeated at least four times in the introduction and several times in the methods section. The authors could revise to avoid repetition. This would make the article concise and easy to follow.** | Edited. See page 3, Lines 76 -77, 82  See page 5, Line 120.  Page 6, Line 158 |
| **3 - The introduction section does not identify a clear gap in the literature that this study is filling and does not clearly describe the objective of this study.** | Revised. See page 5, Line 124 - 126 |
| **5 - Please clarify on the study design - currently the design is described as "preposttest". The authors proceed to mention pretest and post test.  Readers might interpret this as having administered a pretest followed by a post test evaluation as is the case when trainings are done. It might be better to describe this as a retrospective abstraction of medical records that were used for a pre- versus post-intervention evaluation. The pre versus post analysis is used to assess the effect of the intervention on some indicators of the effectiveness of a refferral system.** | Edited.  See the Title – page 1, Line 4  See page 2, Line 40  See page 5, Line 124 - 126 |
| **6 - Study setting section is very long  and repeats concepts already discussed in the introduction section. The details provided here about KNH should be those that are relevant to this evaluation and should be focussed on orthopedics/trauma.** | Edited. See pages 7 and 8 Lines 163 -180 |
| **7 - The section headed " Data management and analysis plan"  - the word "plan" should be removed.**  **This section should clearly explain how data was analyzed. "Descriptive analysis was done using frequency distribution" - probably the authors meant that descriptive analysis was done by calculating frequencies for categorical variables and measures of central tendency/dispersion for continous variables. The statements about inferential analysis and logistic regression are also vague. Please in this section be clear about how the pre- versus post-intervention analysis was conducted - what was compared with what and which statistical tests were used. For logistic regression - what was the outcome of interest? What covariates were used and how was the final model selected?** | Edited. The word “plan” removed. See page 11 Line 232.  Edited. See pages 11  Lines 236 - 244 |
| **8 - Results - it is not clear from table 2 & 4 what the logistic regression results mean and what was the outcome of interest and what was the measure of association and whether the presented results are adjusted or not.** | For table 2, the meaning of the results is on pages 13 and 14, Lines 273 -295. These are adjusted for sex and education. The measure of association is the odds ratio. See page 13 Lines 300.  For table 4, the meaning of the results is on pages 15 and 16, Lines 315 – 324. |
| 9 - Discussion - The first paragraph of this section ought to state what the research question was and what was found in this study. However, the authors start this section by discussing socio-demographic characteristics. In my thinking, this study sought to determine the effect of the implementation of the referral guidelines on categories of new admissions, such as walk-ins. The authors should delve into their main findings concerning this question, describe what they found, what other people found concerning the same question and the implications of their findings.  If this approach is adopted, all the paragraphs headed "Sociodemographic characteristics of the sample population" should be deleted.  The authors could then refine the discussion headed effect of refferral guidelines.  The listed recommendations should be deleted and summarized in a statement the conclusions. | This is an interventional study and therefore a comparison of key socio-demographic characteristics is important to give the readers a preview of sample profile and comparability between the two study populations. This also allows us to how to adjust for the measures of association.  See the comment response above regarding the interventional study/quasi-experimental study  Noted.  Deleted and incorporated in conclusions. See page 27 – 28, Lines 566 – 593. |
| Journal Requirements: |  |
| Please review your reference list to ensure that it is complete and correct. If you have cited papers that have been retracted, please include the rationale for doing so in the manuscript text, or remove these references and replace them with relevant current references. Any changes to the reference list should be mentioned in the rebuttal letter that accompanies your revised manuscript. If you need to cite a retracted article, indicate the article’s retracted status in the References list and also include a citation and full reference for the retraction notice. | Noted |
| Reviewer #1: All comments have been addressed | Noted |
| Reviewer #1: The author has endeavored to address all comments and the manuscript is ready for publication. The language is now more scientific and the methodology section reads well. | Noted |
| Reviewer #3: |  |
| The study titled "Effect of enforcement of referral guidelines on patterns of orthopedic and trauma admissions to Kenyatta National Hospital Kenya: pre-posttest study design" aimed to evaluate the impact of enforcing referral guidelines on the patterns of orthopedic admissions at Kenyatta National Hospital (KNH) in Kenya. The enforcement of referral guidelines was implemented on July 1, 2021, with the goal of reducing walk-in admissions. | Noted. |
| There is very little about trauma admissions in this paper as it seems to focus on the Orthopedic department. For a global audience that understands trauma to be more inclusive than simply orthopedics, I would suggest that the authors remove “trauma” from the title. Or, they could include all admissions involving trauma (craniotomy, laparotomy, thermal injuries, etc.) at KNH. | Edited. See page 1, Line 3 |
| The study employed a pre- and post-intervention design, comparing data from 459 charts before and 446 charts after the enforcement of national referral guidelines. The authors do not discuss the actual numbers of presentations and admissions during this time period (which would be much more interesting), but instead elected to sample charts during each of the time periods. The findings revealed that the enforcement of referral guidelines to limit walk-ins led to a significant decrease in walk-in admissions (from 54.9% to 45.1%). Additionally, non-trauma orthopedic admissions doubled (from 12.0% to 22.4%), and there was a notable increase in admissions through the Outpatient Clinic and Corporate Outpatient Clinic. The study also observed a decline in emergency admissions and an increase in elective admissions, with the increase in elective cases primarily driven by an increase in female admissions with active insurance cover, tertiary education, non-trauma-related conditions, and older age groups. | Noted. The purpose of the study was to see whether enforcement of referral guidelines had reduced the proportion of walks in, so decided to have have random sampling to answer the question. However, we also looked at the trends and it did show decline in number of admissions in general post intervention period. This decline, however, was not sustained from 2022 due to laxity in enforcement of the guideline. However, this component was not part of the current study objectives but could be considered as a follow up study to this. |
| It could be interesting for the hospital to look at impact of the enforcement of their policies; however, I do not believe the results reflect the conclusions. The authors have highlighted the enforcement as a positive development. They have limited walk-ins by enforcing a policy to limit walk-ins. There is very little that is novel or interesting to others. However, what they have demonstrated from their results is that access to care is increasingly difficult with the implementation of guidelines. Those who are able to access care now are more likely to be insured, have higher education, and be older. In other words, they have a higher socioeconomic status and can manage the broken system at KNH. So instead of making the system more accessible and appropriate, these guidelines have increased disparities and limited access to Kenyans from all walks of life. | Thanks for the observation. I have edited the conclusion accordingly. See pages 2 and 3, Lines 53 – 57. Also, see pages 26 and 27, Lines 554 – 562. |
| The policy implementation did not increase the number of referral letters. Instead, it increased discrimination to reduce the number of non-insured, younger patients of lower socioeconomic status (with education as a marker). This could be an interesting conclusion to look at the effects of implementation of policy and would be worthy of publication. | I have edited the conclusion accordingly. See pages 2 and 3, Lines 53 – 57. Also, see pages 26 and 27, Lines 554 – 562. |
| The authors blame others for the congestion at KNH without appreciating that delays in care within KNH also lead to delays in discharges and congestion. The high occupancy rates only tell part of the story. What is the length of stay? How quickly are patients operated on (number and percentage within 24 hours)? There appears to be an underlying assumption that no admission to KNH is justified. For example, to turn someone away and require a referral letter from another facility when there is a threat to life or limb, seems inappropriate. | Page 4, Lines 94 - 97 I did mention the issue of bypassing lower level health facilities as one of the challenges. The other challenges like delays in discharges (discharges in) and delays in treatment also play a role. This study was focusing on patient referrals and the effect of enforcement of national referral guidelines on this.  Admissions to KNH are justified subject to compliance of national referral guidelines.  Unfortunately this study did not seek to measure the severity score of orthopedic admissions. |
| This statement, "This coupled with inadequate staffing levels results in low nurse-patient ratio of 1:10 that compromises not only the quality of nursing care given to patients but also the ability of KNH to effectively perform its statutory obligations” should be supported with evidence/reference. | Three references added. See page 5, Line 113 |
| The authors comment about gender disparities at MTRH. They could also mention the gender disparities that persist outside of the public institutions. At Tenwek Hospital, gender disparities were attributable to delays in definitive care (perhaps because women had more difficulty accessing care at public institutions), but once they arrived to the hospital, the gender disparities in care disappeared suggesting equitable care and inequitable access to care (Otoki K, et al. Gender Disparities in Complications, Costs, and Mortality After Emergency Gastrointestinal Surgery in Kenya. Journal of Surgical Research. 2024 Mar 1;295:846-52.). | Edited. See page 24, Line 489 - 491 |
| The authors mention burn referrals in their references. If the study is examining trauma admissions, patients with burn injuries should be included. If not, trauma should be removed from the title and throughout the manuscript to only focus on orthopedics. For Kenya, some context about the lack of a functional burns referral system may be helpful (Hunter MA, et al. Referral patterns of burn injury in rural Kenya. Journal of Burn Care & Research. 2021 May 1;42(3):454-8.) | Edits done. Trauma removed from the title. See page 1, Line 3. |
| The authors mention PCEA Kikuyu. That is an excellent point about how the majority of orthopedics care is delivered by faith-based institutions. The authors should comment about these hospitals provide the majority of care (Parker RK, et al. Surgical training throughout Africa: a review of operative case volumes at multiple training centers. World journal of surgery. 2020 Jul;44:2100-7.) and most cases happen outside of referral centers (Chokotho L, Jacobsen KH, Burgess D, Labib M, Le G, Lavy CB, Pandit H. Trauma and orthopaedic capacity of 267 hospitals in east central and southern Africa. The Lancet. 2015 Apr 27;385:S17.) | Edited. Page 20, Lines 416 – 418. |
| The authors should comment on the ethics of turning away patients who have arrived for care. For example, if there is an open fracture and the institution is delaying washout by refusing care, that significantly impacts patient outcomes. | Well. This was data was not collected to determine to what extent genuine emergency cases were actually turned away and therefore I am unable to give an objective comment on this. |
| Regarding clarity and consistency, the manuscript would benefit from a thorough revision. For example, the term "pre-posttest study design" is somewhat unconventional; "pre-post intervention study" might be more standard.  Additionally, the use of terms like "enforcement of referral guidelines" and "enforcement of the national referral guidelines" should be consistent throughout the paper.  Additionally, the manuscript requires careful proofreading for grammatical errors and formatting inconsistencies. For example, there are instances of missing spaces between words, inconsistent use of punctuation, and variations in the formatting of headings and subheadings. | Edited. See page 1, Line 4.  Edited throughout the manuscript. "enforcement of the national referral guidelines used throughout” the manuscript  Formatted. |
| The authors seem to be dismissive of the previous reviewers' comments.  Each of these comments should be addressed and the specific place that they are addressed should be included (page/line number) and the specific changes should be included in the comments to reviewers (not just referring to the tracked changes). | See reviewers 1 comment above. Also see reviewers 2 comments in the previous round of revisions. All comments including the Academic Editors comments were addressed. All comments in the current review are all new comments.  The addition of page/line included in this round of comments/revisions. |
